# Diketopiperazine Alkaloids and Bisabolene Sesquiterpenoids from *Aspergillus versicolor* AS-212, an Endozoic Fungus Associated with Deep-Sea Coral of Magellan Seamounts

**DOI:** 10.3390/md21050293

**Published:** 2023-05-10

**Authors:** Yu-Liang Dong, Xiao-Ming Li, Xiao-Shan Shi, Yi-Ran Wang, Bin-Gui Wang, Ling-Hong Meng

**Affiliations:** 1 CAS and Shandong Province Key Laboratory of Experimental Marine Biology, Institute of Oceanology, Chinese Academy of Sciences, Nanhai Road 7, Qingdao 266071, China; dongyuliang@qdio.ac.cn (Y.-L.D.); lixmqd@qdio.ac.cn (X.-M.L.); shixs@hbnu.edu.cn (X.-S.S.); wangyiran@qdio.ac.cn (Y.-R.W.); 2 Laboratory of Marine Biology and Biotechnology, Qingdao National Laboratory for Marine Science and Technology, Wenhai Road 1, Qingdao 266237, China; 3 University of Chinese Academy of Sciences, Yuquan Road 19A, Beijing 100049, China; 4 Center for Ocean Mega-Science, Chinese Academy of Sciences, Nanhai Road 7, Qingdao 266071, China

**Keywords:** diketopiperazine, *Aspergillus versicolor*, deep-sea coral, endophytic fungus, antimicrobial activity

## Abstract

Two new quinazolinone diketopiperazine alkaloids, including versicomide E (**2**) and cottoquinazoline H (**4**), together with ten known compounds (**1**, **3**, and **5**–**12**) were isolated and identified from *Aspergillus versicolor* AS-212, an endozoic fungus associated with the deep-sea coral *Hemicorallium* cf. *imperiale*, which was collected from the Magellan Seamounts. Their chemical structures were determined by an extensive interpretation of the spectroscopic and X-ray crystallographic data as well as specific rotation calculation, ECD calculation, and comparison of their ECD spectra. The absolute configurations of (−)-isoversicomide A (**1**) and cottoquinazoline A (**3**) were not assigned in the literature reports and were solved in the present work by single-crystal X-ray diffraction analysis. In the antibacterial assays, compound **3** exhibited antibacterial activity against aquatic pathogenic bacteria *Aeromonas hydrophilia* with an MIC value of 18.6 μM, while compounds **4** and **8** exhibited inhibitory effects against *Vibrio harveyi* and *V. parahaemolyticus* with MIC values ranging from 9.0 to 18.1 μM.

## 1. Introduction

Marine-derived fungi living under extreme survival conditions are considered as abundant sources of structurally diverse and biologically active compounds [1,2]. In the deep-sea habitats, seamounts are regarded locations for a wide variety of current-topography interactions and biophysical coupling which have large biomass and higher biodiversity than their surrounding deep-sea floors [3,4]. Endozoic fungi surviving in deep-sea seamounts are a promising new source to mining bioactive secondary metabolites owing to their unique habitats. To date, only three papers investigating bioactive secondary metabolites of fungi derived from deep-sea seamounts have been published [5,6,7]. Therefore, a study on the chemical diversity of deep-sea seamount-derived endozoic fungi is warranted.

The species in the fungal genus *Aspergillus*, especially *A. versicolor*, is widely distributed in various habitats (marine, terrestrial, and symbiotic sources) and possesses the ability to produce diversified bioactive secondary metabolites such as diketopiperazine alkaloids [8,9], peptides [10], xanthones [9,11], and sesquiterpenes [12]. Most of these metabolites are described to exhibit a variety of bioactivities, including antifungal [9], antitumor [10,11], and neuroprotective activities [12].

**Figure 1:**
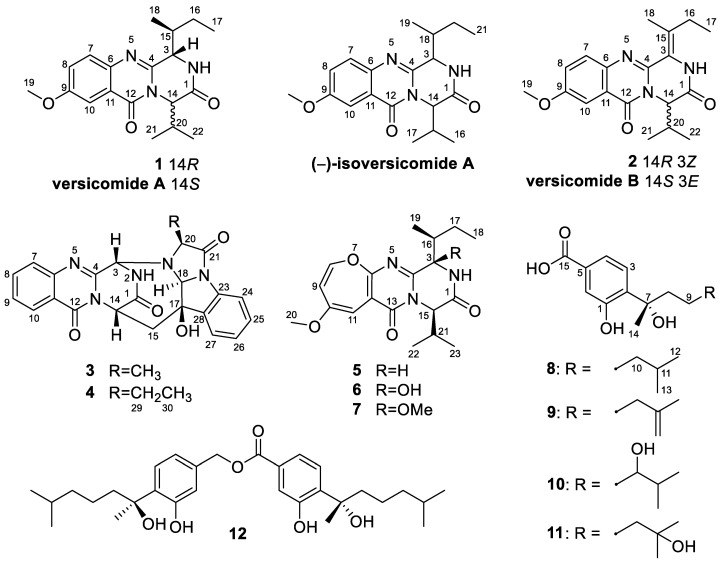
Structures of compounds **1**–**12**, versicomides A and B, and (−)-isoversicomide A.

In our continuous efforts to explore bioactive metabolites from deep-sea seamount-derived fungi [5,6,7], chemical investigation of the endozoic fungus *Aspergillus versicolor* AS-212 associated with the deep-sea coral, *Hemicorallium* cf. *imperiale*, which was collected from the Magellan Seamounts in the Western Pacific Ocean was carried out due to its unique HPLC profiles. As a result, two new quinazolinone diketopiperazine alkaloids, namely, versicomide E (**2**) and cottoquinazoline H (**4**), together with five known related analogs (**1**, **3**, **5**–**7**) as well as four known bisabolene derivatives (**8**–**11**) and a bisabolene dimer (**12**), have been isolated and identified. Herein, we report the isolation and structure elucidation as well as the antimicrobial activities of compounds **1**–**12** (Figure 1).

## 2. Results and Discussion

### 2.1. Structure Elucidation of the Isolated Compounds

**Figure 2:**
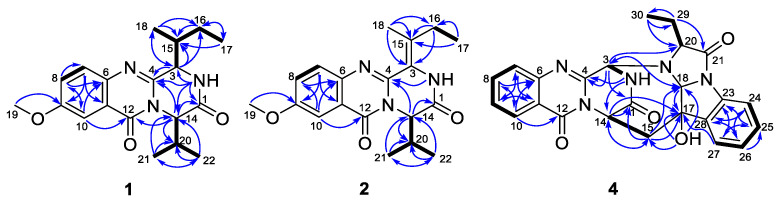
Key COSY (bold lines) and HMBC (blue arrows) correlations for compounds **1**, **2**, and **4**.

**Figure 3:**
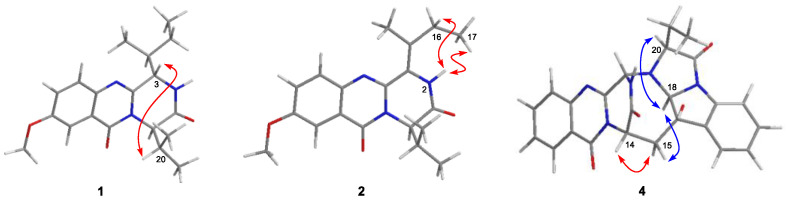
Key NOE correlations for compounds **1**, **2**, and **4** (red lines: *β*-orientation; blue lines: *α*-orientation).

**Figure 4:**
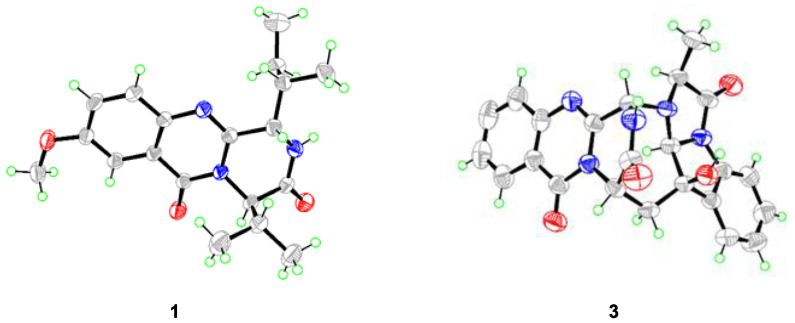
X-ray crystal structures of compounds **1** and **3**.

Compound **1** was isolated as colorless crystals, and the molecular formula was established as C_19_H_25_N_3_O_3_ by analysis of the HRESIMS data. The ^1^H and ^13^C NMR of **1** (DMSO-*d*_6_, Table 1) extremely resembled those of versicomide A, a quinazoline-containing compound isolated from the crab-derived fungus *Aspergillus versicolor* XZ-4 which was collected from hydrothermal vent [8]. Further analysis of the 2D NMR spectra (Figure 2) indicated the same planar structure of **1** as that of versicomide A (Figure 1). However, a strong NOE cross-peak of H-3/H-20 was in favor of the structure with the 3S*- and 14R*-relative configuration rather than a 3S* and 14S* configuration (Figure 3). Single-crystal X-ray diffraction analysis with Cu Kα radiation further demonstrated its structure and absolute configurations (Figure 4). A Flack parameter of 0.0(2) enabled the definition of its absolute configuration as 3*S*, 14*R*, and 15*S*, indicating that **1** was the 14-epimer of versicomide A.

Compound **1** was initially treated as a new quinazoline alkaloid during the preparation of this manuscript, while Tasdemir and co-workers recently reported a new quinazoline-containing diketopiperazine (−)-isoversicomide A from the deep-sea sediment-derived fungus *Aspergillus versicolor* PS108-62 [13]. Notably, compound **1** shared the same planar structure and virtually similar optical rotation value (${\left[\alpha \right]}_{D}^{25}$ −30 vs. ${\left[\alpha \right]}_{D}^{20}$ −25) as that of (−)-isoversicomide A, in which the stereogenic centers at C-3 and C-14 showed the same relative configurations with that of compound **1**. However, the configuration at C-18 on the short flexible aliphatic chain and the absolute configuration of (−)-isoversicomide A were not assigned due to the limited sample available [13]. Considering their similar rotation values and same relative configuration at C-3 and C-14, we assumed that compound **1** and (−)-isoversicomide A are the same compound. As the reported evidence to determine the absolute configuration of versicomide A does not seem entirely solid and in view of the highly similar NMR data of those isomers with multi-chiral centers, it is necessary to clarify the absolute configuration of **1**. The results from the X-ray diffraction analysis of compound **1** unambiguously determined its absolute configuration as 3*S*, 14*R*, and 15*S*. This is likely the first time the configuration of isoleucine in a quinazoline-containing diketopiperazine skeleton with a Val-Ile cyclic dipeptide moiety was unambiguously defined by X-ray crystallography analysis.

Versicomide E (**2**) was obtained as a colorless amorphous solid with the molecular formula C_19_H_23_N_3_O_3_ based on the HRESIMS data. Its NMR data (CDCl_3_, Table 1) were similar to those of **1**, which indicated that **2** possessed the same quinazoline backbone as **1**. The obvious difference was the absence of signals for two methines at *δ*_C_ 58.1/*δ*_H_ 4.70 (CH-3) and *δ*_C_ 36.1/*δ*_H_ 2.62 (CH-15) in the NMR spectra of **1**, whereas additional resonances corresponding to a tetra-substituted double bond at *δ*_C_ 121.3 (C-3) and *δ*_C_ 135.0 (C-15) were found in that of **2** (CDCl_3_, Table 1), which were further confirmed by COSY and HMBC correlations (Figure 2). The geometry of the double bond between C-3 and C-15 was determined as Z-configuration by key NOE correlations from NH-2 (*δ*_H_ 8.02) to H-16 (*δ*_H_ 2.29) and H_3_-17 (*δ*_H_ 1.15) (Figure 3). Compound **2** has the same planar structure as that of versicomide B (Figure 1), which was also isolated from hydrothermal vent crab-derived fungus *Aspergillus versicolor* XZ-4 by Wu and co-workers in 2017 [8], with the exception of the geometry of the double bond at C3(15) (*Z* in **2** vs. *E* in versicomide B) and the absolute configuration of C-14 (*R* in **2** vs. *S* in versicomide B) as well. To clarify the stereochemistry of compound **2**, calculations of specific rotation (SR) were carried out for 14*R*-**2** and 14*S*-**2**, and the calculated SR value for 14*R*-**2** (+59.8) at CAM-B3LYP/TZVP level was compatible with the experimental SR value ${\left[\alpha \right]}_{D}^{25}$ +112.0 (*c* 0.08, MeOH), contrary to that of versicomide B (${\left[\alpha \right]}_{D}^{20}$ −23.4) [8], which allowed the assignment of absolute configuration of C-14 in **2** as 14*R* (Table S2). To further verify the absolute configuration of C-14 in **2**, the time-dependent density functional (TDDFT)-ECD calculation was performed on 14*R*-**2** and 14*S*-**2** at the CAM-B3LYP/TZVP level in Gaussian 09. The experimental curve matched that of the calculated ECD spectrum for 14*R*-**2** and also assigned the absolute configuration of C-14 in **2** as 14*R* (Figure S27).

Compound **3** was obtained as colorless prisms and was identified as cottoquinazoline A by comparing its NMR data (measured in DMSO-*d*_6_, Table S3) with those previously reported in the literature [10]. Cottoquinazoline A is a 16-nor analog of the known fumiquinazoline D and was first isolated from a marine-derived fungal strain of *A. versicolor* (MST-MF495) by Capon and co-workers in 2009, with a partial stereostructure assigned [10]. Considering the complexity of the structure of **3** and the presence of many stereoisomers, it is important to clarify the assignment of the absolute configurations of **3** [10,14]. Fortunately, a suitable crystal of **3** was picked out from DMSO–MeOH (1:1) and subjected to X-ray crystallographic analysis to assign its absolute configurations of the stereogenic centers in **3** as 3*S*, 14*S*, 16*R*, 17*S*, and 19*S* (Figure 4).

**Figure 5:**
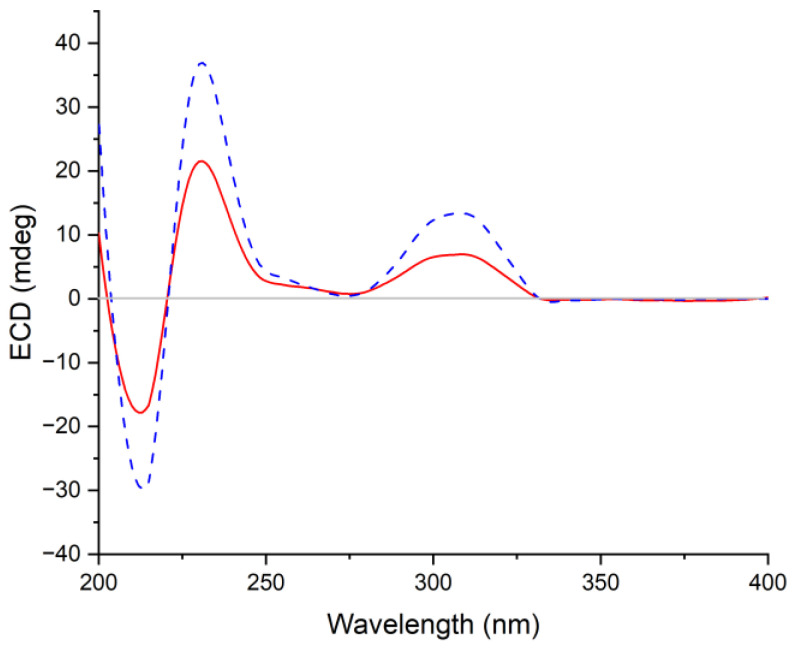
Comparison of the experimental ECD spectra of compounds **3** (in red) and **4** (in blue) in CH_3_OH.

Cottoquinazoline H (**4**) was obtained as a colorless amorphous solid. Its molecular formula was established as C_24_H_21_N_5_O_4_ by HRESIMS, with one CH_2_ unit more than that of **3**. Discreet comparisons of the NMR data (DMSO-*d*_6_, Table 2) and UV absorptions with **3** suggested that they shared the same core scaffold. However, the methyl substitution at C-20 in **3** was replaced by an ethyl group in **4**, as evidenced by the appearance of an additional methylene group resonating at *δ*_C_ 21.0 and *δ*_H_ 1.90/1.99 (CH_2_-29) in the NMR spectra of **4** (DMSO-*d*_6_, Table 2). Additionally, the chemical shift of C-20 was deshielded downfield from *δ*_C_ 63.2 in **3** to *δ*_C_ 68.1 in **4**. The COSY and HMBC correlations verified the above deduction (Figure 2). The relative configuration of **4** was also deduced from the analysis of NOESY experiments. The NOE cross-peaks from H-20 and H-15α to H-18 revealed the cofacial orientation of these groups (Figure 3). Given that the stereochemistry of co-isolated compound **3** was determined by X-ray diffraction analysis as well as their similar NMR chemical shifts and virtually identical experimental ECD curves (Figure 5), the absolute configurations of all chiral carbons in **4** were established as 3*S*, 14*S*, 17*R*, 18*S*, and 20*S*.

In addition to compounds **1**–**4**, three related quinazolinone diketopiperazine alkaloids, namely, versicoloids A and B (**5** and **6**) [9], and chrysopiperazine A (**7**) [15], as well as five known bisabolene derivatives (**8**–**12**) including sydonic acid (**8**) [16], (*S*)-(+)-11-dehydrosydonic acid (**9**) [17], (−)-10-hydroxysydonic acid (**10**) [18], hydroxysydonic acid (**11**) [16], and peniciaculin B (**12**) [18] were also identified and isolated from the fungus *A. versicolor* AS-212, which were determined by the comparison of their NMR data and those previously described in the literature. Structurally, (−)-isoversicomide A (**1**) might be a plausible biosynthetic precursor that undergoes the transformation of the benzene ring to the oxepine ring to generate versicoloid A (**5**) [19], which provides the basis for the biosynthetic origins of versicoloid A.

### 2.2. Antimicrobial Assays

The antimicrobial activity evaluation of all the isolated compounds was performed against human pathogenic bacterium (*Escherichia coli*), marine-derived aquatic pathogenetic bacteria (*Aeromonas hydrophila*, *Edwardsiella ictarda*, *Micrococcus luteus*, *Pseudomonas aeruginosa*, *Vibrio harveyi*, *V. parahemolyticus*, *V. vulnificus*), and plant-pathogenic fungi (*Colletotrichum gloeosporioides*, *Curvularia spicifera*, *Epicoccum sorghinum*, *Fusarium oxysporum*, *F. proliferatum*, and *Penicillium digitatum*) (Table 3). In the antimicrobial screening, compounds **4** and **8** exhibited potent inhibitory activity against the aquatic pathogenic bacterium *V. parahaemolyticus* with MIC values of 9.0 and 15.0 μM, while compounds **8** and **9** showed inhibitory activity against the aquatic pathogenic bacterium *V. harveyi* with the MIC values of 15.0 and 15.2 μM. In addition, compounds **3** and **4** displayed a broad spectrum of antimicrobial activity against most of the tested strains, with the MIC values ranging from 9.0 to 74.6 μM. The bisabolene derivatives (**8**–**12**) mainly exhibited activities against *M. luteus*, *V. harveyi*, and *V. parahaemolyticus,* with MIC values ranging from 15.0 to 121.2 μM. However, neither the quinazoline-containing diketopiperazine derivatives (**1** and **2**) nor the oxepine-containing diketopiperazine analogs (**5**–**7**) showed any activity against all the tested pathogenic bacteria. These data suggested that the 16-*nor*-methyl fumiquinazoline alkaloids generally showed higher antimicrobial activity than that of quinazolinone alkaloids (**3** and **4** vs. **1** and **2**) and the oxepine congeners (**5**–**7**). A comparison of the antimicrobial results of **3** and **4** revealed that different substituent groups at C-20 could influence the inhibitory potency against the pathogenic bacteria. Concerning bisabolene derivatives, the antimicrobial results revealed that compound **12**, a dimeric bisabolene analog, showed weaker antimicrobial activities than that of the monomeric bisabolenes (**8**–**11**) against *M. luteus* and *V. harveyi*. In addition, hydroxylation at C-10 or C-11 likely decreased the activity against *V. harveyi, V. parahaemolyticus,* and *C. gloeosporioides* (**8** vs. **10** and **11**).

The above results showed that compounds **4**, **8**, and **9** were found to be efficient in suppressing the growth of aquatic pathogenic bacteria *V. parahaemolyticus* and *V. harveyi*. To a great degree, the endozoic fungus *A. versicolor* AS-212 which is associated with the deep-sea coral *Hemicorallium* cf. *imperiale* may provide a chemical defense to help its host to fight off the aquatic pathogenic bacteria by producing an array of antimicrobial secondary metabolites.

## 3. Experimental Section

### 3.1. General Experimental Procedures

The general experimental procedures, apparatus, and solvents/reagents used in this work were the same as those described in our previous reports [5,6,7].

### 3.2. Fungal Material

The endophytic fungus *Aspergillus versicolor* AS-212 associated with deep-sea coral, *Hemicorallium* cf. *imperial*, was collected from the Magellan Seamounts (depth 1420 m) in May 2018. By comparing its ITS region sequence with that of *A. versicolor* (accession no. MT582751.1) in the GenBank database, the sequence data of strain AS-212 were identical (100%) to those of *A. versicolor* and subsequently uploaded in GenBank with accession no. OP009765.1. The fungus AS-212 has been conserved at the Key Laboratory of Experimental Marine Biology, Institute of Oceanology, Chinese Academy of Sciences (IOCAS).

### 3.3. Fermentation, Extraction, and Isolation

The fungal strain AS-212 was cultivated on potato dextrose agar (PDA) plates at 28 °C for 7 days to generate spores. The fresh mycelia were transferred into 1 L Erlenmeyer flasks, each containing 300 mL potato-dextrose broth (PDB) medium, which was reported in our previous publication [5], and fermented under static conditions for 30 days at room temperature. After 30 days of incubation, a total of 33 L cultures were filtered and collected to separate the broth and mycelia. The broth was adequately extracted three times with EtOAc, while the mycelia were mechanically crushed and then extracted three times with 80% volume aqueous acetone. Acetone was removed in vacuo to afford an aqueous solution, which was successively extracted with EtOAc. Based on their virtually similar TLC and HPLC profiles (Figure S26), both EtOAc extracts from broth and mycelia were combined and evaporated under a vacuum to render the EtOAc extract (61 g).

The EtOAc extract was subjected to vacuum liquid chromatography (VLC) eluted with petroleum ether (PE)–EtOAc gradient (20:1 to 1:1, *v*/*v*) and then CH_2_Cl_2_–MeOH (20:1 to 1:1, *v*/*v*) to afford nine fractions (Frs. 1–9). Fr. 4 (2.3 g) was fractionated by reverse-phase column chromatography (CC) with a MeOH–H_2_O gradient (from 10:90 to 100:0) to afford nine subfractions (Frs.4.1–4.9). Fr. 4.2 was directly purified by semi-preparative HPLC (Elite ODS-BP, 5µm; 10 × 250 mm; 80% MeOH–H_2_O, 2.5 mL/min) to yield compound **6** (3.0 mg, *t*_R_ = 17 min). Fr. 4.4 (16 mg) was further purified by prep. TLC (plate: 20 × 20 cm, developing solvents: PE–EtOAc, 2:1) and by Sephadex LH-20 (MeOH) column to afford **7** (3.1 mg). Fr. 4.5 was purified by CC over Sephadex LH-20 chromatography (MeOH) and then by semi-preparative HPLC (85% MeOH–H_2_O, 2.5 mL/min) to give compound **1** (6.6 mg, *t*_R_ = 14 min). Fr. 4.6 (75 mg) was fractionated by CC on Sephadex LH-20 column (MeOH) to yield five subfractions Frs.4.6.1–4.6.5. Fr. 4.6.5 (20 mg) was further purified by prep. TLC (developing solvents: DCM–MeOH, 20:1) and by Sephadex LH-20 (MeOH) to afford compound **9** (7.7 mg). Fr. 4.7 (36 mg) was directly purified by prep. TLC (developing solvents: CH_2_Cl_2_–EtOAc, 3:1) and by Sephadex LH-20 (MeOH) to afford compound **8** (4.0 mg). Fr. 4.8 (67 mg) was purified by CC on silica gel eluting with CH_2_Cl_2_–MeOH gradient (from 200:1 to 50:1) to obtain compound **12** (4.3 mg). Fr. 5 (3.4 g) was separated by reversed-phase CC using step-gradient elution with MeOH–H_2_O (from 10:90 to 100:0) to yield seven subfractions (Frs. 5.1–5.7). Fr. 5.2 (241 mg) was fractionated by CC on silica gel eluting with CH_2_Cl_2_–MeOH gradient (from 150:1 to 20:1) and then purified on Sephadex LH-20 (MeOH) to afford compounds **10** (11.3 mg) and **11** (4.3 mg). Fr. 5.4 (126 mg) was fractionated by CC on Sephadex LH-20 (MeOH) and further purified by semi-preparative HPLC (70% MeOH–H_2_O, 2.5 mL/min) to afford compound **5** (6.3 mg, *t*_R_ = 22 min). Fr. 5.6 was chromatographed via a Sephadex LH-20 column (MeOH) and then by semi-preparative HPLC (78% MeOH-H_2_O, 2.5 mL/min) to afford compound **2** (6.7 mg, *t*_R_ = 20 min). Fr. 7 (5.8 g) was fractionated by reverse-phase CC with a MeOH–H_2_O gradient (from 10:90 to 100:0) to yield five subfractions (Frs. 7.1–7.5). Fr. 7.5 (328 mg) was applied to silica gel CC eluted with CH_2_Cl_2_/MeOH to give nine subfractions (Frs. 7.5.1–7.5.9). Fr.7.5.7 (36 mg) was purified by semi-preparative HPLC (45% MeCN–H_2_O, 2.5 mL/min) to provide compounds **3** (4.3 mg, *t*_R_ = 9 min) and **4** (3.2 mg, *t*_R_ = 12 min).

 

(−)-Isoversicomide A (**1**): colorless crystals; mp 197−199 °C; ${\left[\alpha \right]}_{D}^{25}$ −30 (*c* 0.10, MeOH); UV (MeOH) *λ*_max_ (log *ε*) 227 (3.47), 277 (3.00), 326 (2.58) nm; ECD (0.52 mM, MeOH) *λ*_max_ (Δ*ε*) 210 (−4.87), 233 (−15.03), 277 (+1.88), 328 (−1.02) nm; for ^1^H and ^13^C NMR data, see Table 1; HRESIMS *m/z* 344.1960 [M + H]^+^ (calcd for C_19_H_26_N_3_O_3_, 344.1969).

 

Versicomide E (**2**): colorless amorphous solid; ${\left[\alpha \right]}_{D}^{25}$ +112 (*c* 0.08, MeOH); UV (MeOH) *λ*_max_ (log *ε*) 221 (3.26), 309 (2.88) nm; ECD (0.59 mM, MeOH) *λ*_max_ (Δ*ε*) 207 (+6.69), 233 (−3.87), 255 (+6.18), 300 (+1.41), 343 (−1.15) nm; for ^1^H and ^13^C NMR data, see Table 1; HRESIMS *m/z* 340.1663 [M − H]^−^ (calcd for C_19_H_22_N_3_O_3_, 340.1667).

 

Cottoquinazoline A (**3**): colorless prisms (MeOH-DMSO 1:1); mp 215−217 °C; ${\left[\alpha \right]}_{D}^{25}$ +160 (*c* 0.10, MeOH); UV (MeOH) *λ*_max_ (log *ε*) 205 (3.39), 227 (3.17), 256 (2.81), 268 (2.74), 280 (2.68), 305 (2.26), 315 (2.14) nm; ECD (0.58 mM, MeOH) *λ*_max_ (Δ*ε*) 211 (−13.16), 230 (+12.22), 308 (+3.60) nm; for ^1^H and ^13^C NMR data, see Table S2.

 

Cottoquinazoline H (**4**): colorless amorphous solid; ${\left[\alpha \right]}_{D}^{25}$ +150 (*c* 0.10, MeOH); UV (MeOH) *λ*_max_ (log *ε*) 205 (3.60), 227 (3.43), 257 (3.11), 270 (3.03), 279 (2.96), 304 (2.52), 317 (2.40) nm; ECD (0.56 mM, MeOH) *λ*_max_ (Δ*ε*) 212 (−22.91), 231 (+21.65), 308 (+7.16) nm; for ^1^H and ^13^C NMR data, see Table 2; HRESIMS *m/z* 444.1663 [M + H]^+^ (calcd for C_24_H_22_N_5_O_4_, 444.1666).

### 3.4. X-ray Crystallographic Analysis of Compounds ***1*** and ***3***

Suitable crystals were picked out to obtain crystallographic data using a Bruker Smart-1000 or Bruker D8 VENTURE CCD diffractometer with Cu Kα radiation (λ = 1.54178 Å). Absorption correction was applied using the program SADABS [20]. The structures were solved by direct methods with the SHELXTL software package [21,22]. All non-hydrogen atoms were refined anisotropically. The absolute structures were determined by refinement of the Flack parameter [23]. The structures were optimized by full-matrix least-squares techniques. Crystallographic data have been deposited with the Cambridge Crystallographic Data Centre with deposition numbers CCDCs 2192654 and 2192653 for **1** and **3**, respectively. Crystal data and structure refinements for **1** and **3** are listed in Table S1.

*Crystal data for compound **1**:* C_19_H_25_N_3_O_3_, F.W. = 343.2, space group P2(1)2(1)2(1), unit cell dimensions a = 13.2384(3) Å, b = 19.9347(4) Å, c = 6.8103(2) Å, V = 1797.26(8) Å^3^, α = β = γ = 90°, Z = 4, d_calcd_ = 1.269 g/cm^3^, crystal dimensions 0.350 × 0.330 × 0.300 mm, μ = 0.702 mm^–1^, F(000) = 736. The 4007 measurements yielded 2826 independent reflections after equivalent data were averaged. The final refinement gave R_1_ = 0.0379 and wR_2_ = 0.0989 [I > 2σ(I)]. Flack parameter = 0.0(2).

*Crystal data for compound **3**:* 2(C_23_H_18_N_5_O_4_)·C_2_OS_2_, F.W. = 960.99, orthorhombic space group C222_1_, unit cell dimensions a = 9.4022(11) Å, b = 25.878(4) Å, c = 19.112(2) Å, V = 4650.2(11) Å^3^, α = β = γ = 90°, Z = 4, d_calcd_ = 1.373 g/cm^3^, crystal dimensions 0.200 × 0.180 × 0.150 mm, μ = 1.612 mm^–1^, F(000) = 1992. The 21,138 measurements yielded 4267 independent reflections after equivalent data were averaged. The final refinement gave R_1_ = 0.0951 and wR_2_ = 0.2851 [I > 2σ(I)]. Flack parameter = 0.145(12).

### 3.5. Antimicrobial Assay

A two-fold serial dilution method using 96-well microtiter plates was applied to evaluating the antimicrobial activities against a panel of aquatic pathogenic bacteria (*Aeromonas hydrophilia* QDIO-1, *Edwardsiella ictarda* QDIO-9, *Micrococcus luteus* QDIO-3, *Pseudomonas aeruginosa* QDIO-4, *Vibrio harveyi* QDIO-7, *V. parahaemolyticus* QDIO-8, and *V. vulnificus* QDIO-10), one human pathogenic bacterium (*Escherichia coli* EMBLC-1), and six plant-pathogenic fungi (*Penicillium digitatum* QDAU-3, *Colletotrichum gloeosporioides* QA-29, *Fusarium oxysporum* QDAU-8, *Curvularia spicifera* QA-26, *Epicoccum sorghinum* QA-20, and *F. proliferatum* QA-28) [24]. The aquatic pathogenic strains and human pathogenic bacterium were provided by IOCAS, while the plant-pathogenic fungi were provided by IOCAS and Qingdao Agricultural University. To assay the antimicrobial activities, DMSO was added to dissolve all isolated compounds and positive control (chloramphenicol and amphotericin B) to prepare a stock solution with a specific concentration.

### 3.6. Specific Rotation and ECD Calculations

General computational procedures were consistent with our previous reports [5,25].

## 4. Conclusions

In conclusion, two new quinazolinone derivatives, versicomide E (**2**) and cottoquinazoline H (**4**), along with ten known compounds (**1**, **3**, and **5**–**12**), were isolated and identified from the deep-sea coral-derived *Aspergillus versicolor* AS-212. This marks the first time that the absolute configurations of all the stereogenic centers in (−)-isoversicomide A (**1**) and cottoquinazoline A (**3**), which were not assigned in the previous literature, were accurately solved in the present work by X-ray crystallographic analysis. Compound **3** exhibited activity against aquatic pathogenic bacteria *A. hydrophilia* with an MIC value of 18.6 μM, while compounds **4** and **8**–**10** exhibited inhibitory effects against *V. harveyi* with MIC values ranging from 15.0 to 28.4 μM. In addition, compounds **4** and **8** exhibited potent inhibitory effects against *V. parahaemolyticus* with MIC values of 9.0 and 15.0 μM, which might have the potential to be developed as leading compounds in discovering aquatic antibiotics.

## Figures and Tables

**Table 1:** ^1^H (500 MHz) and ^13^C (125 MHz) NMR Spectroscopic Data for Compounds **1** and **2**.

Compound 1 ^a^				Compound 2 ^b^		
No.	*δ*_C_, Type	*δ*_H_ (Mult, *J* in Hz)	HMBC (From H to C)	*δ*_C_, Type	*δ*_H_ (Mult, *J* in Hz)	HMBC (From H to C)
1	168.0, C			167.1, C		
2		8.40, br s	1, 3, 4, 14		8.02, br s	4
3	58.1, CH	4.70, d (1.7)	4, 15, 16, 18	121.3, C		
4	149.3, C			144.3, C		
6	141.4, C			141.7, C		
7	129.5, CH	7.61, d (8.9)	9, 11	129.3, CH	7.62, d (8.9)	9, 11
8	124.9, CH	7.45, dd (8.9, 2.9)	6, 7, 10	125.1, CH	7.35, dd (8.9, 2.9)	6
9	158.5, C			159.0, C		
10	106.8, CH	7.52, d (2.9)	6, 8, 12	106.4, CH	7.65, d (2.9)	6, 8
11	120.9, C			120.8, C		
12	160.7, C			160.9, C		
14	60.9, CH	4.93, d (8.7)	1, 4, 12, 20	61.2, CH	5.33, dd (8.3, 1.5)	1, 4, 20
15	36.1, CH	2.62, m		135.0, C		
16	23.5, CH_2_	1.34, m	15, 17	27.8, CH_2_	2.29, m	3, 15
17	12.9, CH_3_	0.86, overlap	15, 16	11.6, CH_3_	1.15, t (7.6)	15, 16
18	15.5, CH_3_	1.13, d (7.2)	3, 15, 16	19.5, CH_3_	2.35, s	3, 15, 16
19	56.2, CH_3_	3.88, s	9	56.0, CH_3_	3.92, s	9
20	30.8, CH	2.26, m		32.2, CH	2.17, m	
21	20.2, CH_3_	0.86, overlap	14, 20, 22	19.8, CH_3_	1.01, d (6.8)	14, 20, 22
22	19.5, CH_3_	1.04, d (6.6)	14, 20, 21	19.0, CH_3_	1.09, d (6.8)	14, 20, 21

^a^ Recorded in DMSO-*d*_6_. ^b^ Recorded in CDCl_3._

**Table 2:** ^1^H (500 MHz) and ^13^C (125 MHz) NMR data for compound **4** (in DMSO-*d*_6_).

Compound 4			
No.	*δ*_C_, Type	*δ*_H_ (Mult, *J* in Hz)	HMBC (From H to C)
1	167.8, C		
2		9.10, d, (5.1)	4, 14
3	65.4, CH	5.22, d, (5.1)	1, 4, 18, 20
4	147.4, C		
6	146.7, C		
7	127.2, CH	7.74, dd, (8.4,1.0)	9, 11
8	134.4, CH	7.84, ddd, (8.4, 7.1, 1.5)	6, 10
9	127.1, CH	7.55, ddd, (8.1, 7.1, 1.0)	7, 11
10	126,1, CH	8.13, dd, (8.0, 1.5)	6, 8, 12
11	120.7, C		
12	159.3, C		
14	53.8, CH	5.27, dd, (5.3, 2.4)	1, 15, 17
15	36.3, CH_2_	3.08, dd, (14.9, 5.3) 2.42, dd, (14.9, 2.4)	17, 18 1, 14
17	73.9, C		
18	79.6, CH	4.87, d (1.8)	3, 17
20	68.1, CH	4.06, m	
21	164.6, C		
23	135.9, C		
24	113.6, CH	7.29, overlap	26, 28
25	129.3, CH	7.29, overlap	23, 27
26	124.3, CH	7.09, ddd (7.6, 4.2, 3.4)	24, 25, 28
27	124.4, CH	7.42, d, (7.6)	17, 25
28	139.6, C	-	
29	21.0, CH_2_	1.90, m 1.99, m	21, 30
30	8.9, CH_3_	1.06, t, (7.4)	20, 29
17-OH		5.35, s	15, 17, 18

**Table 3:** The antimicrobial activities of compounds **1**–**12** (MIC, μM) ^a^.

Strains	1	2	3	4	5	6	7	8	9	10	11	12	Positive Control
*A. hydrophila*	-	-	18.6	-	-	-	-	-	-	-	-	-	6.2 ^b^
*E. coli*	-	-	-	72.2	-	-	-	-	-	-	-	-	6.2 ^b^
*M. luteus*	-	-	74.6	36.1	-	-	-	-	-	-	-	-	3.1 ^b^
*V.* *harveyi*	-	-	37.3	18.1	-	-	-	15.0	15.2	28.4	-	-	3.1 ^b^
*V. parahaemolyticus*	-	-	37.3	9.0	-	-	-	15.0	121.2	113.5	113.5	64.0	3.1 ^b^
*V. vulnificus*	-	-	74.6	72.2	-	-	-	-	-	-	-	-	3.1 ^b^
*C. spicifera*	93.3	187.7	74.6	72.2	-	170.1	-	-	-	-	-	-	1.1 ^c^
*C. gloeosporioides*	186.6	-	74.6	72.2	89.1	170.1	164.5	120.3	121.2	-	-	-	2.2 ^c^

^a^ (-) = MIC > 200 μM; Positive control: ^b^ Chloromycetin; ^c^ amphotericin B.

## Data Availability

Not applicable.

## Supplementary Materials

The following supporting information can be downloaded at: https://www.mdpi.com/article/10.3390/md21050293/s1, Figures S1–S27: The analyzed data of MS, 1D and 2D NMR spectra of compounds **1**–**4**, crystal packing of compounds **1** and **3**, HPLC analysis of mycelia extract, broth extract, and compounds **1**–**12** of *Aspergillus versicolor* AS-212, and experimental and calculated ECD spectra of compound **2** at the CAM-B3LYP/TZVP level. Table S1: Crystal data and structure refinement for compounds **1** and **3**. Table S2: Calculated specific rotation values at 589.44 nm for the enantiomers 14*R*-**2** and 14*S*-**2** at the CAM-B3LYP/TZVP level. Table S3: ^1^H and ^13^C NMR spectroscopic data for compound **3**.
