# Supplementary material for: Diketopiperazine Alkaloids and Bisabolene Sesquiterpenoids from Aspergillus versicolor AS-212, an Endozoic Fungus Associated with Deep-Sea Coral of Magellan Seamounts
Source: Mar Drugs. 2023 May 10;21(5):293. doi: 10.3390/md21050293 (PMC10224163; doi:10.3390/md21050293)
Supplement: Supplementary file 1 [file marinedrugs-21-00293-s001.zip › marinedrugs-2355186-supplementary.pdf]

Supplementary Material

Diketopiperazine Alkaloids and Bisabolene Sesquiterpenoids from *Aspergillus versicolor* AS-212, an Endozoic Fungus Associated with Deep-Sea Coral of Magellan Seamounts

Content

Figure S1. HRESI mass spectrum of compound **1**;

Figure S2. ^{1}H NMR (500 MHz, DMSO-d_6) spectrum of compound **1**;

Figure S3. ^{13}C NMR (125 MHz, DMSO-d_6) and DEPT spectra of compound **1**;

Figure S4. COSY spectrum of compound **1**;

Figure S5. HSQC spectrum of compound **1**;

Figure S6. HMBC spectrum of compound **1**;

Figure S7. NOESY spectrum of compound **1**;

Figure S8. HRESI mass spectrum of compound **2**;

Figure S9. ^{1}H NMR (500 MHz, $CDCl_3$) spectrum of compound **2**;

Figure S10. ^{13}C NMR (125 MHz, $CDCl_3$) and DEPT spectra of compound **2**;

Figure S11. COSY spectrum of compound **2**;

Figure S12. HSQC spectrum of compound **2**;

Figure S13. HMBC spectrum of compound **2**;

Figure S14. NOESY spectrum of compound **2**;

Figure S15. ^{1}H NMR (500 MHz, DMSO-d_6) spectrum of compound **3**;

Figure S16. ^{13}C NMR (125 MHz, DMSO-d_6) and DEPT spectra of compound **3**.

Figure S17. HRESI mass spectrum of compound **4**

Figure S18. ^{1}H NMR (500 MHz, DMSO-d_6) spectrum of compound **4**;

Figure S19. ^{13}C NMR (125 MHz, DMSO-d_6) and DEPT spectra of compound **4**.

Figure S20. COSY spectrum of compound **4**;

Figure S21. HSQC spectrum of compound **4**;

Figure S22. HMBC spectrum of compound **4**;

Figure S23. NOESY spectrum of compound **4**;

Figure S24. Crystal packing of compound **1** at 297(2) K;

Figure S25. Crystal packing of compound **3** at 297(2) K;

Figure S26. HPLC analysis of mycelia extract, broth extract, and compounds **1**−**12** of *Aspergillus versicolor* AS-212;

Figure S27. Experimental and calculated ECD spectra of compound **2** at the CAM-B3LYP/TZVP level;

Table S1. Crystal data and structure refinement for compounds **1** and **3**;

Table S2. Calculated specific rotation values at 589.44 nm for the enantiomers 14*R*-**2** and 14*S*-**2** at the CAM-B3LYP/TZVP level;

Table S3. ^{1}H and ^{13}C NMR spectroscopic data for compound **3**.

Figure S1. HRESI mass spectrum of compound **1**.

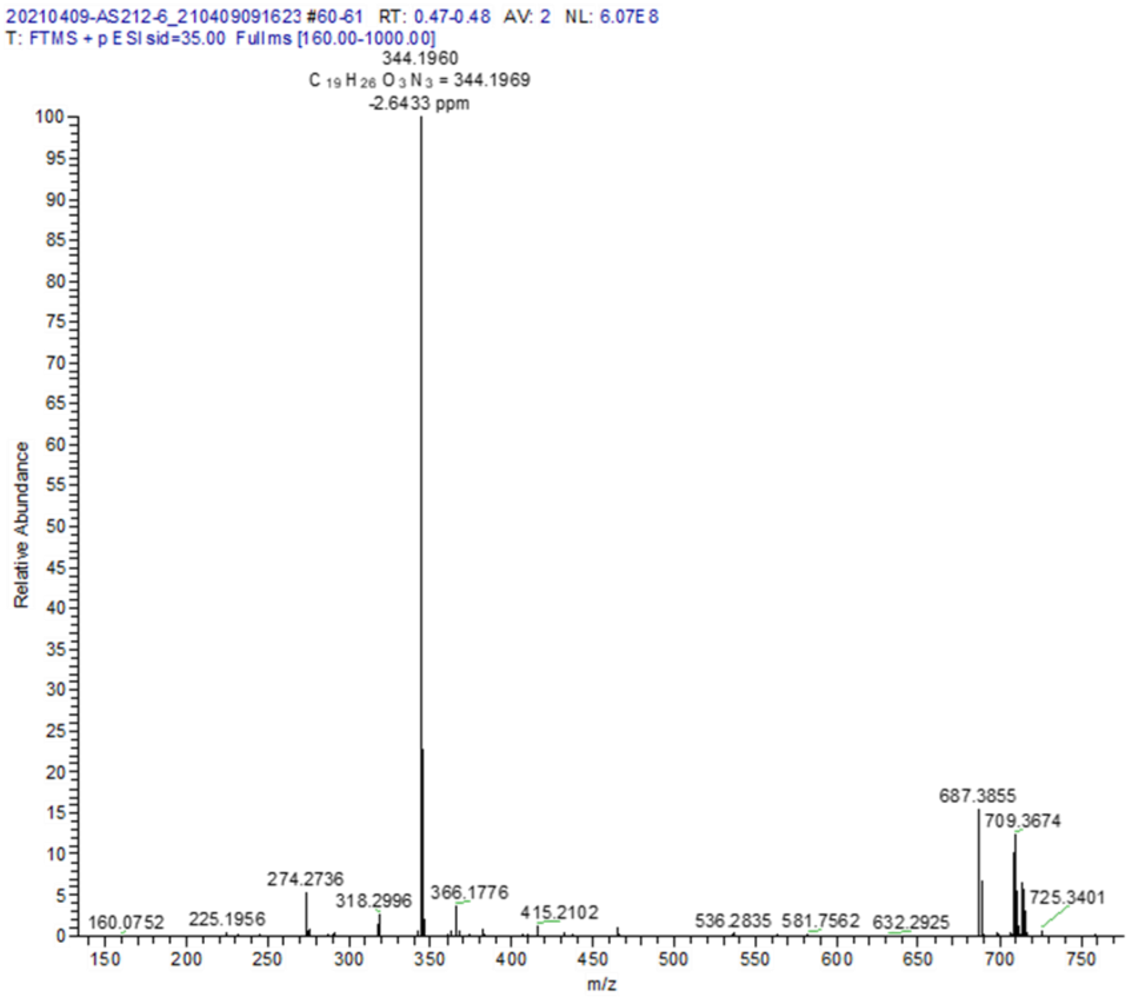

Figure S2. ^{1}H NMR (500 MHz, DMSO-d_6) spectrum of compound **1**.

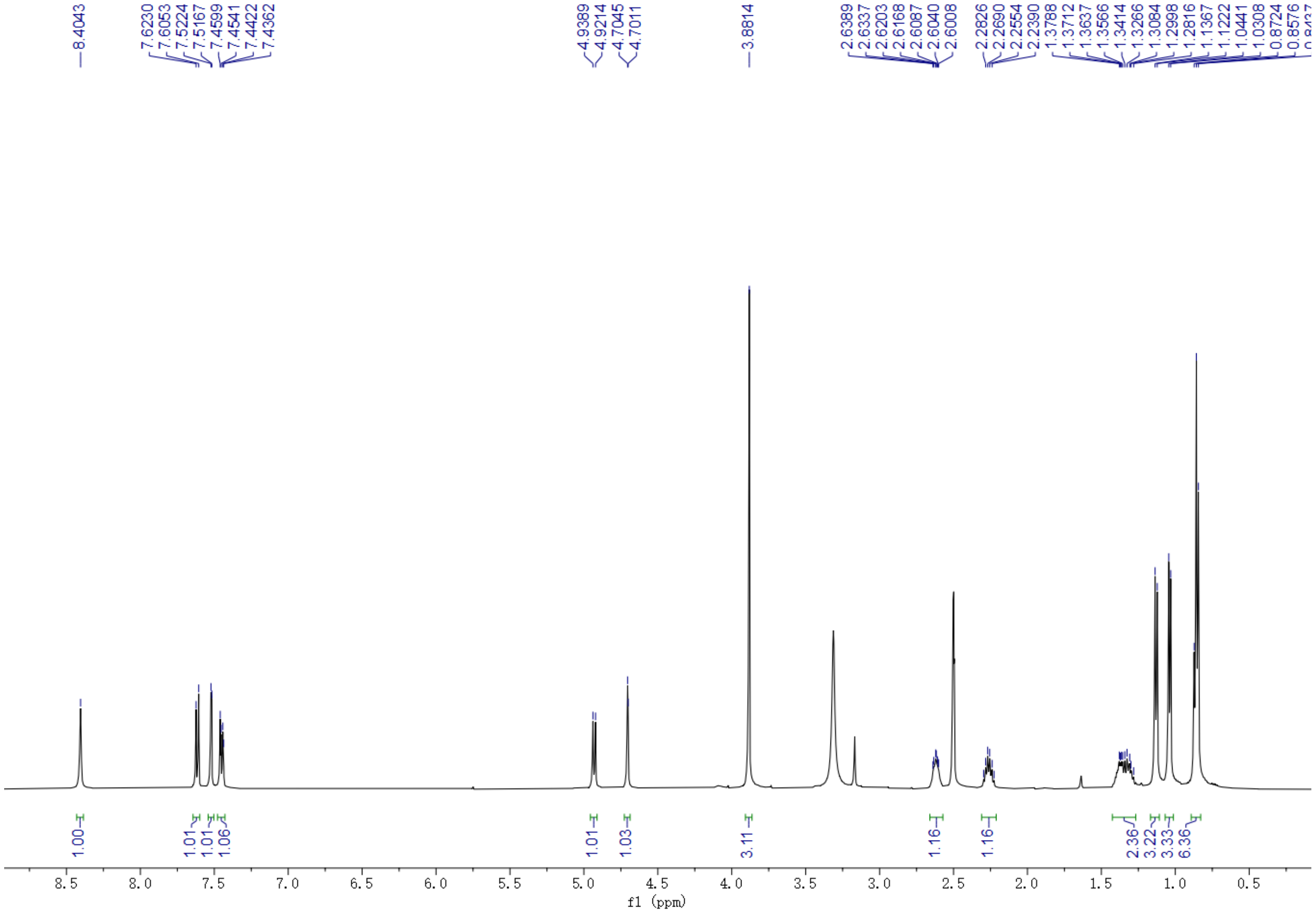

Figure S3. ^{13}C NMR (125 MHz, DMSO-d_6) and DEPT spectra of compound **1**.

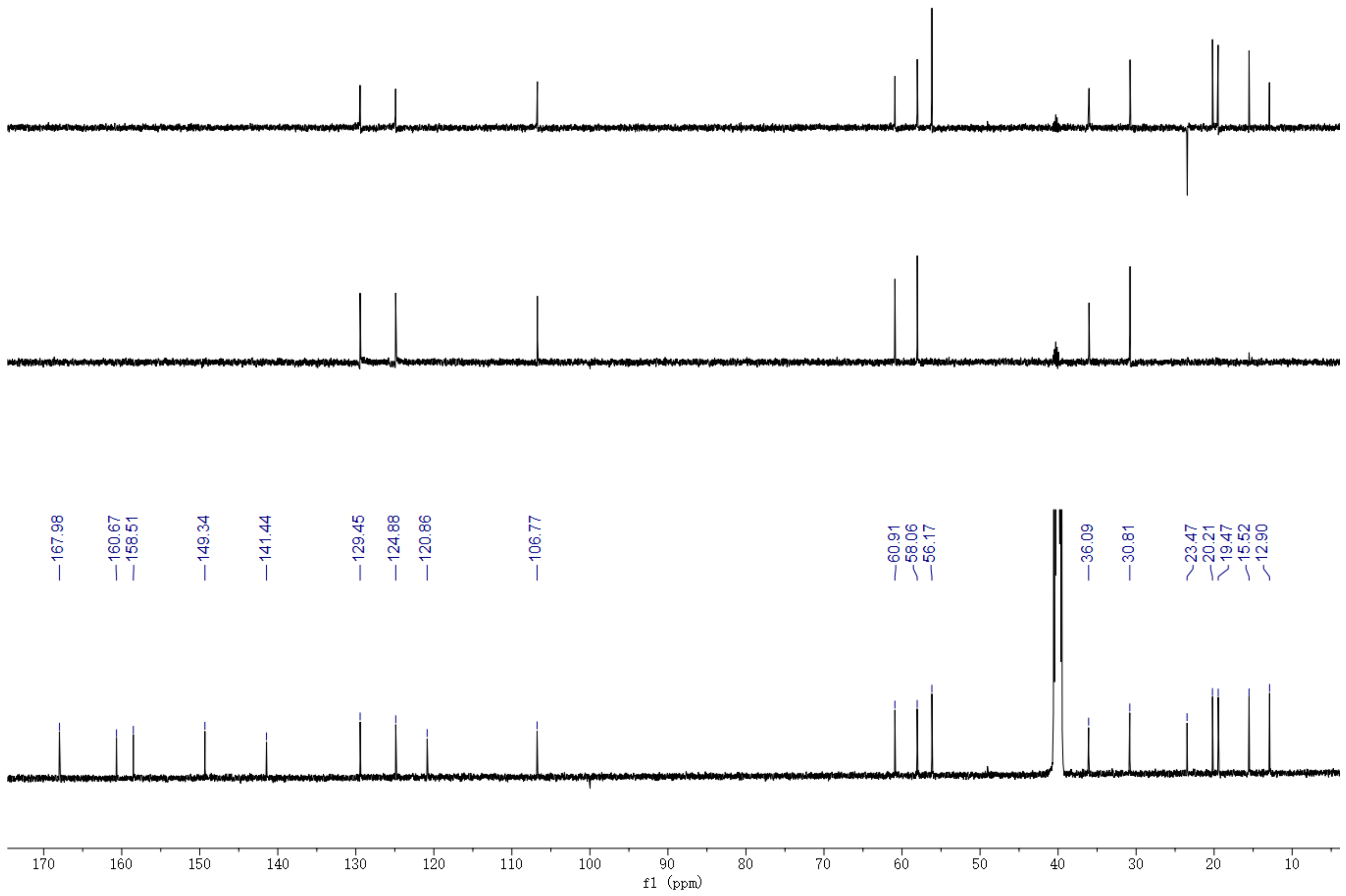

Figure S4. COSY spectrum of compound **1**.

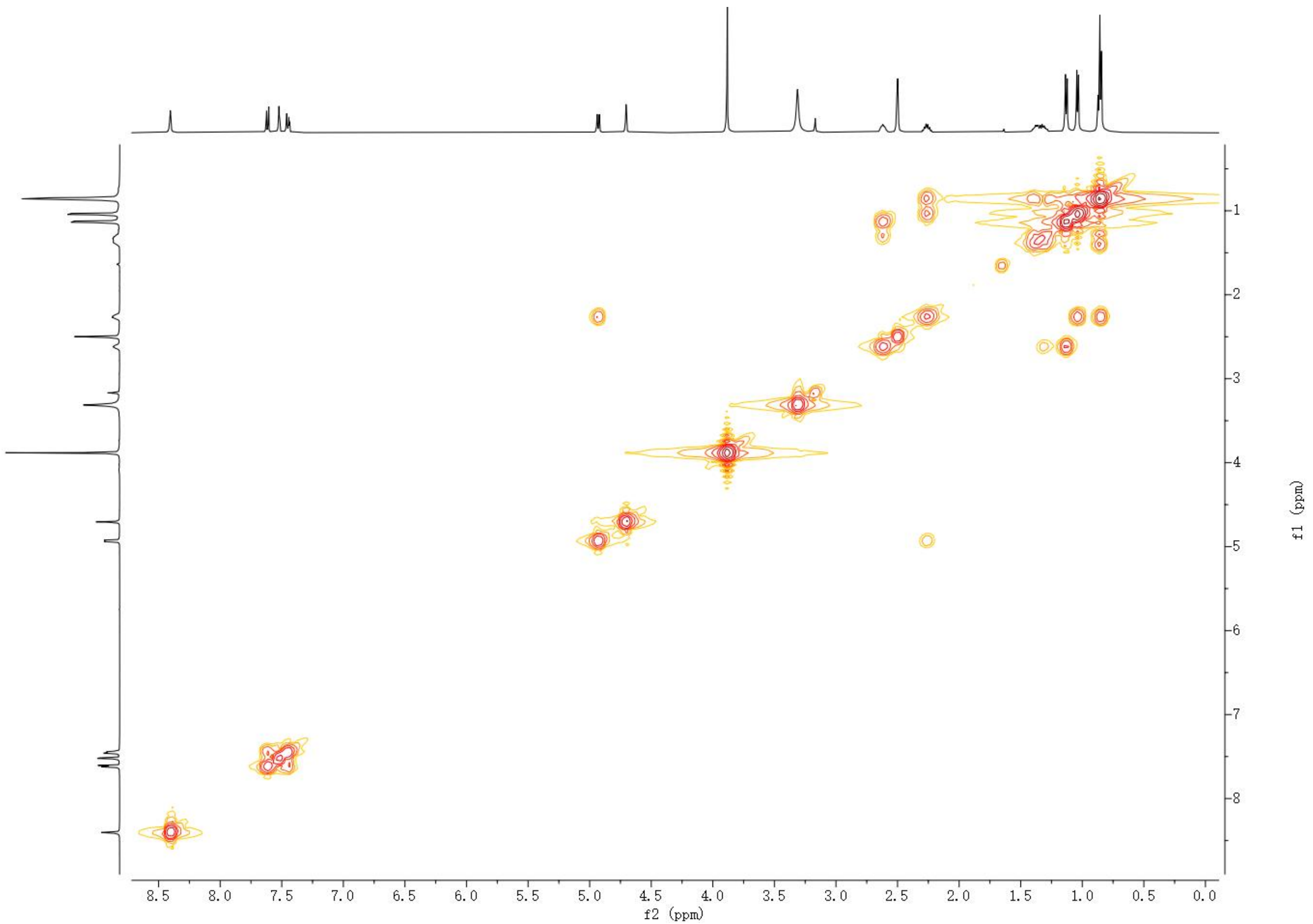

Figure S5. HSQC spectrum of compound **1**.

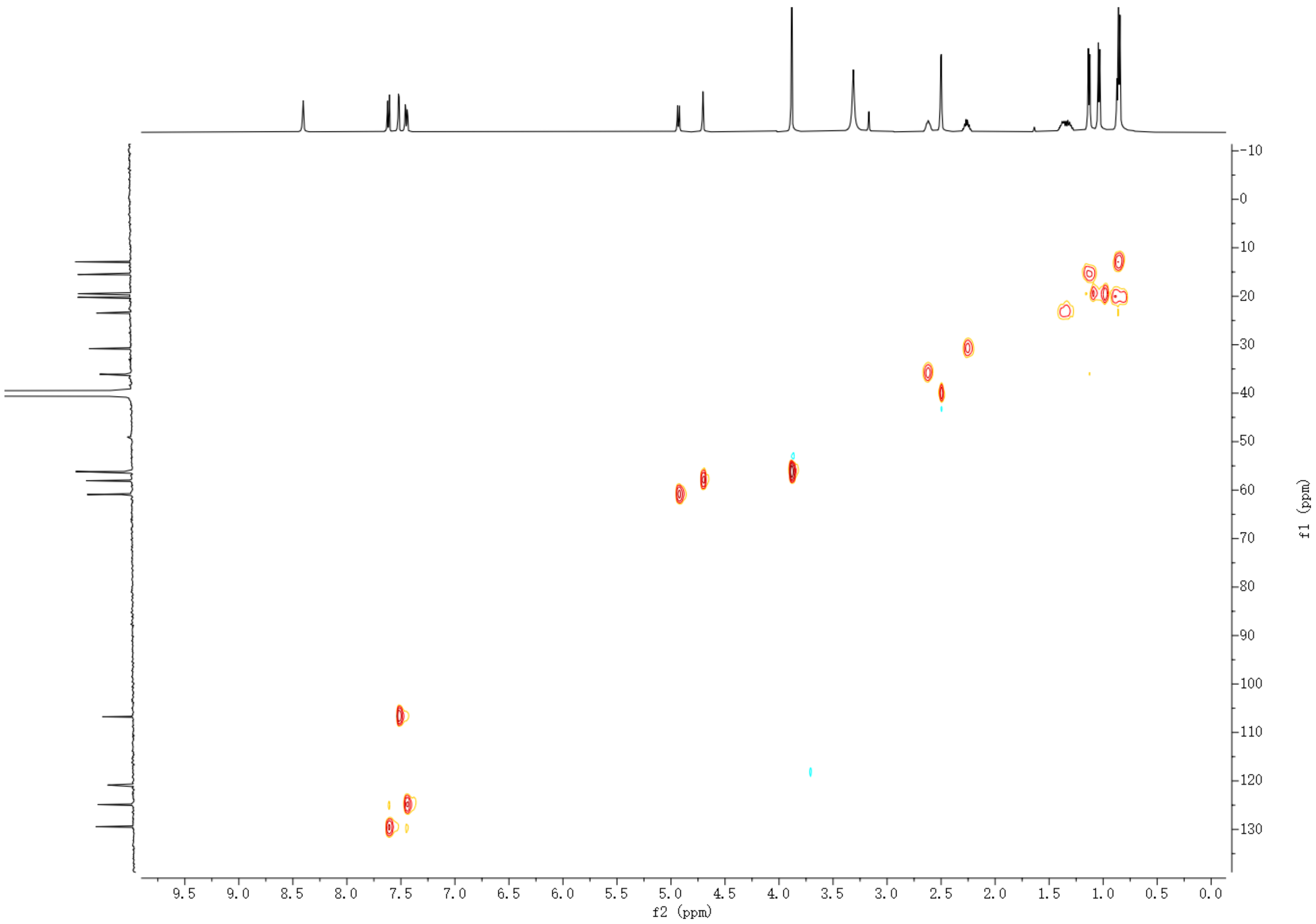

Figure S6. HMBC spectrum of compound **1**.

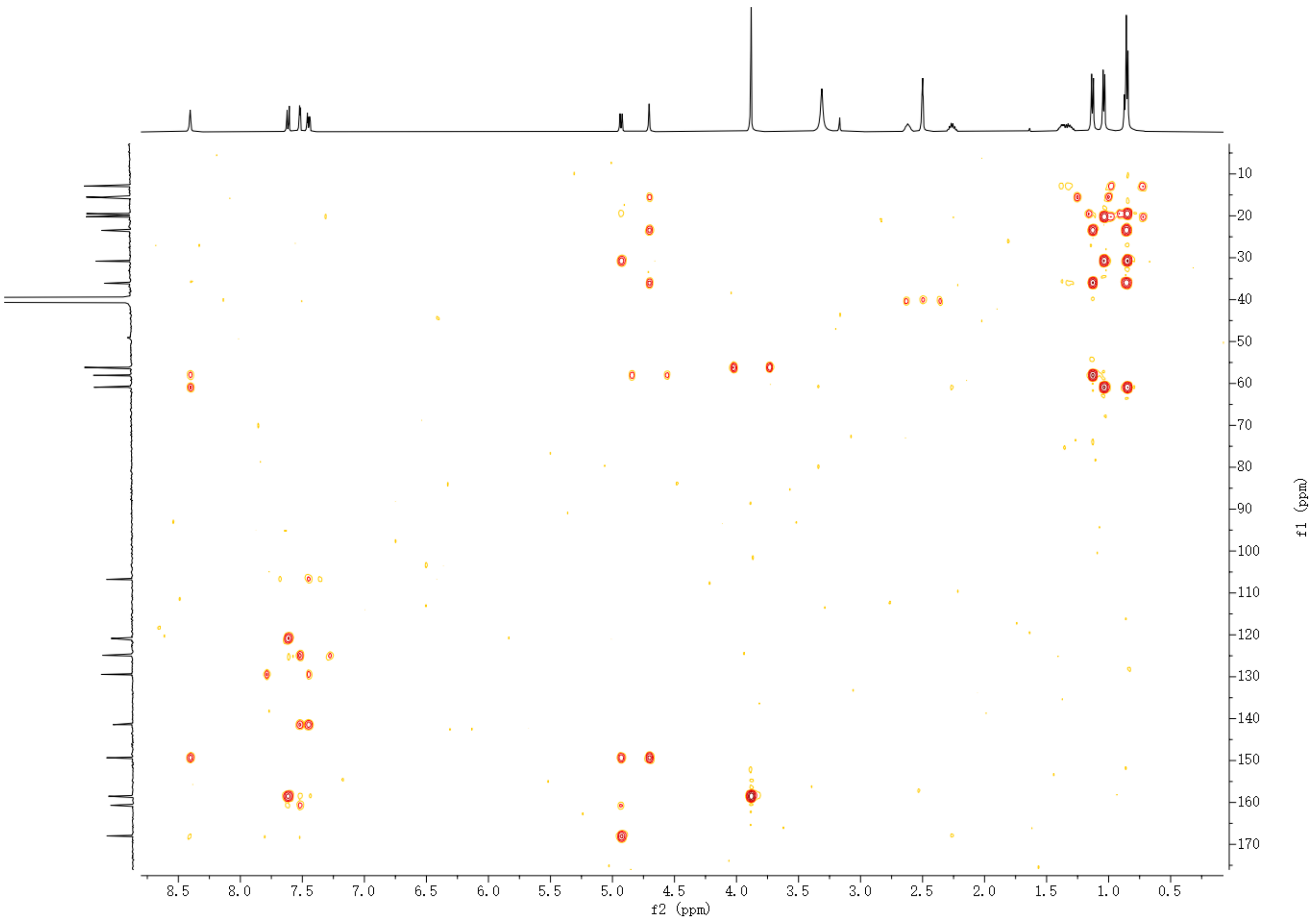

Figure S7. NOESY spectrum of compound **1**.

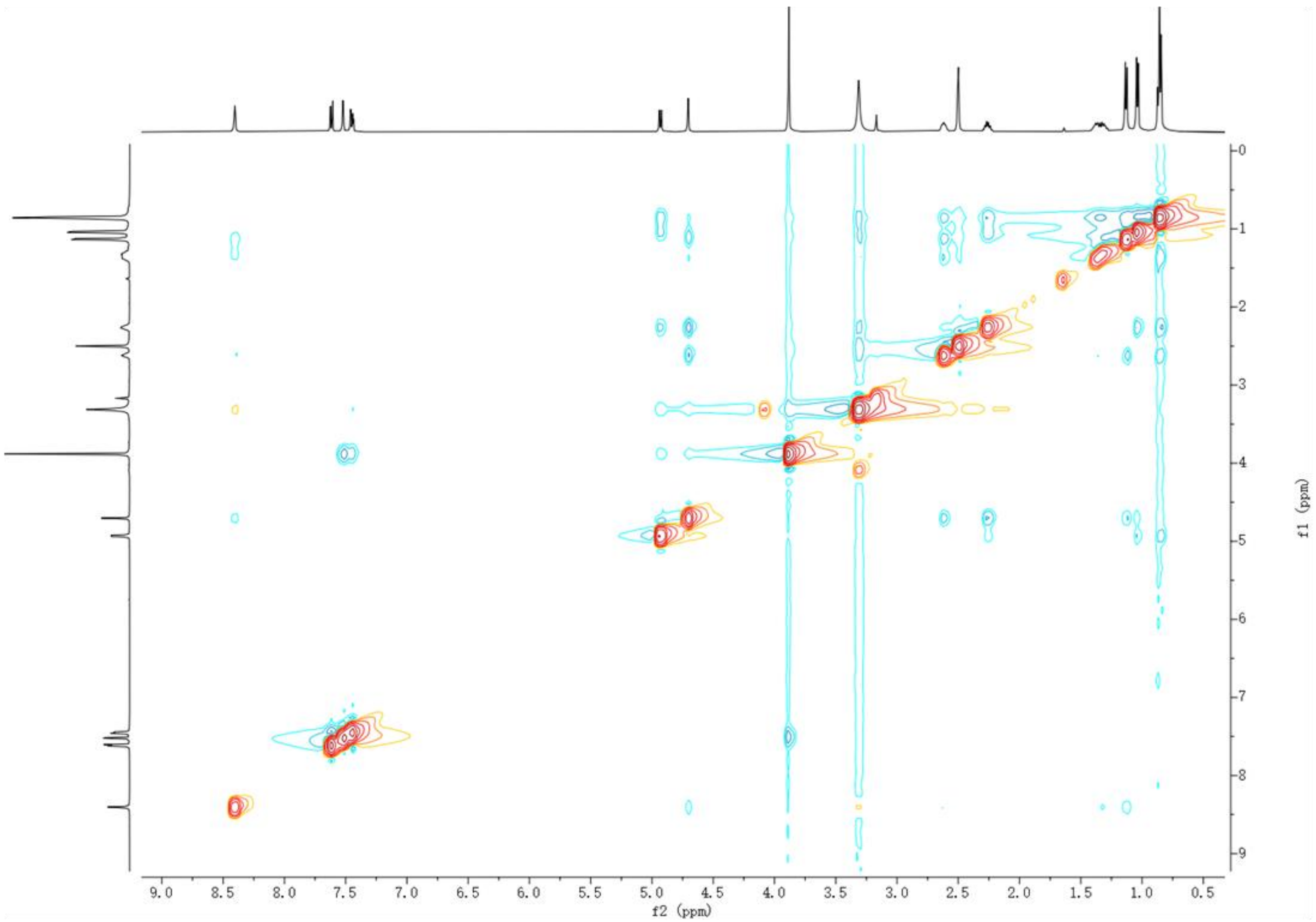

Figure S8. HRESI mass spectrum of compound **2**.

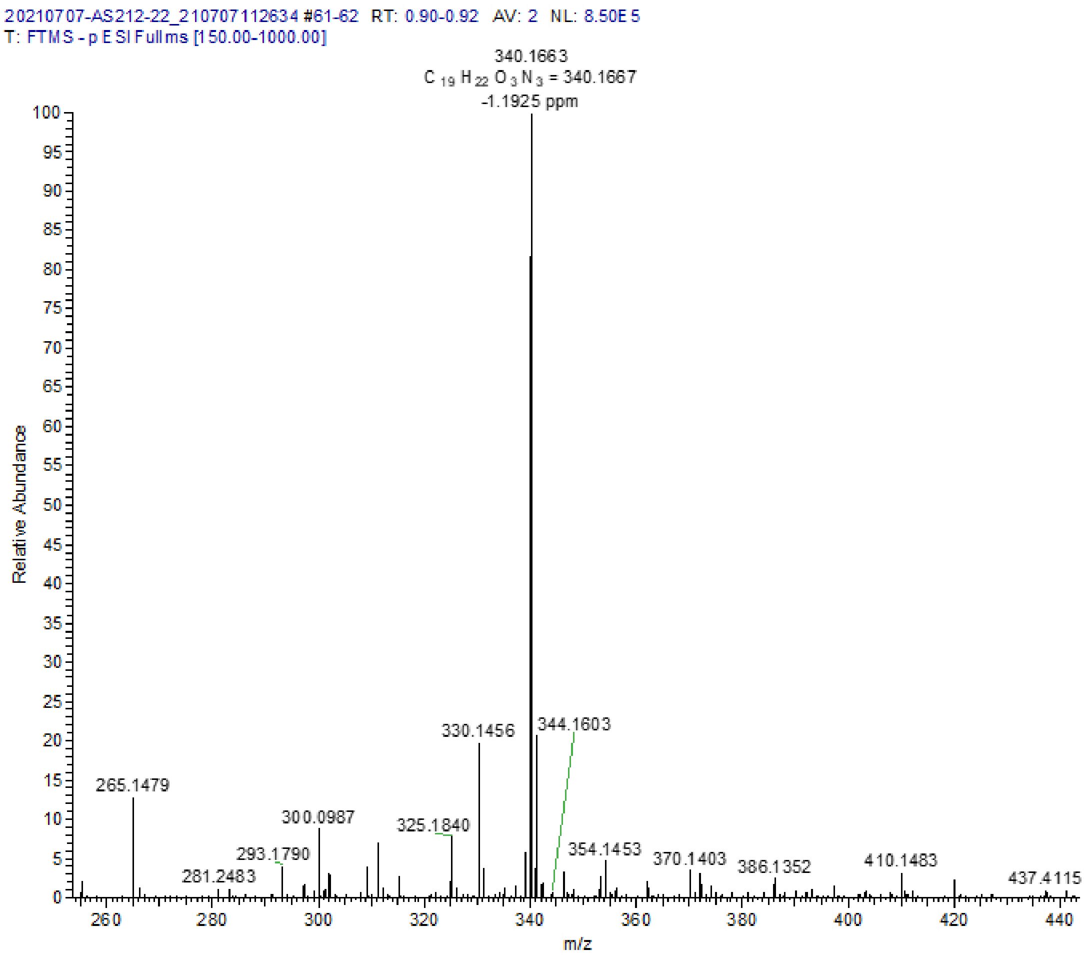

Figure S9. ^{1}H NMR (500 MHz, $CDCl_3$) spectrum of compound **2**.

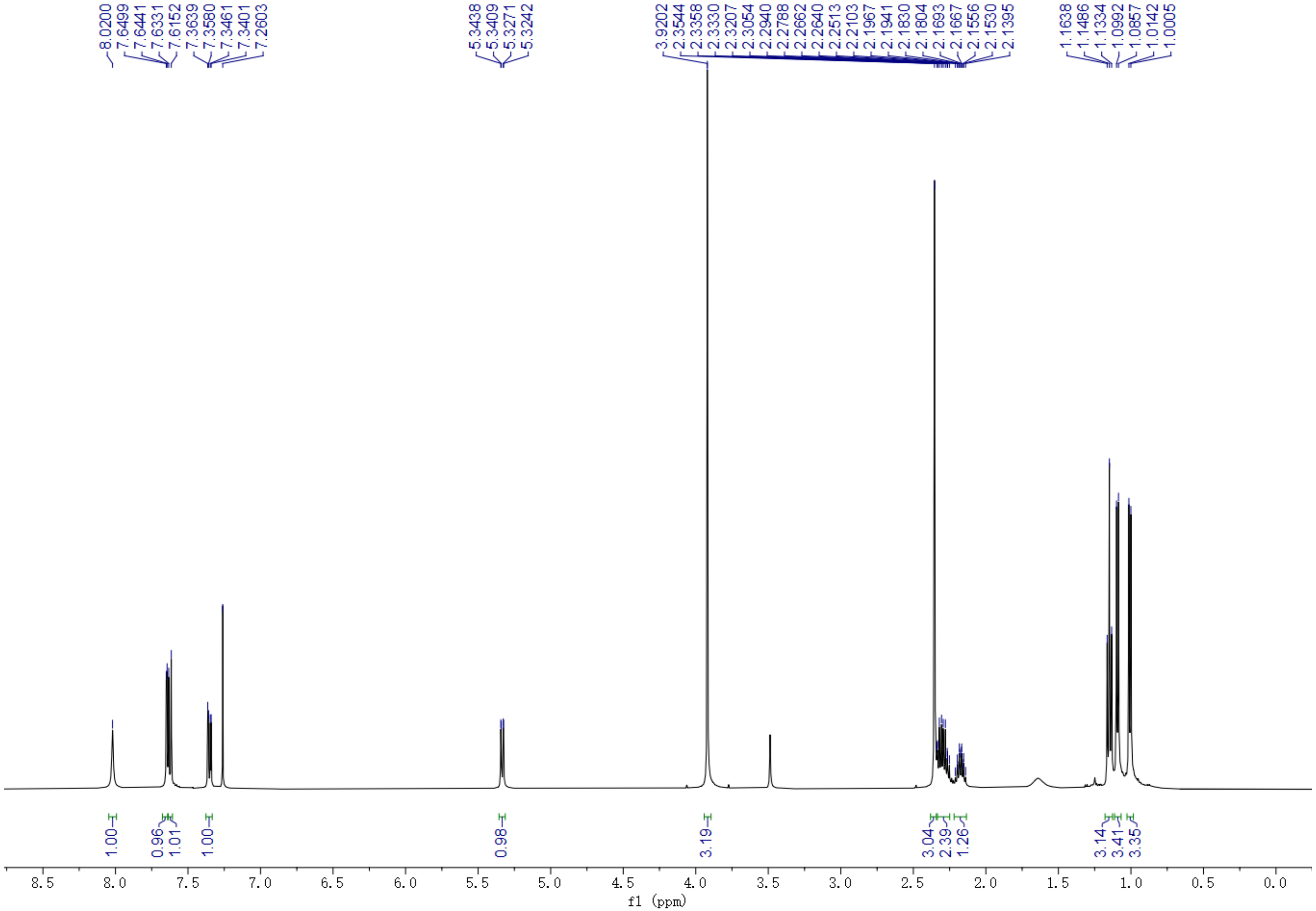

Figure S10. ^{13}C NMR (125 MHz, $CDCl_3$) and DEPT spectra of compound **2**.

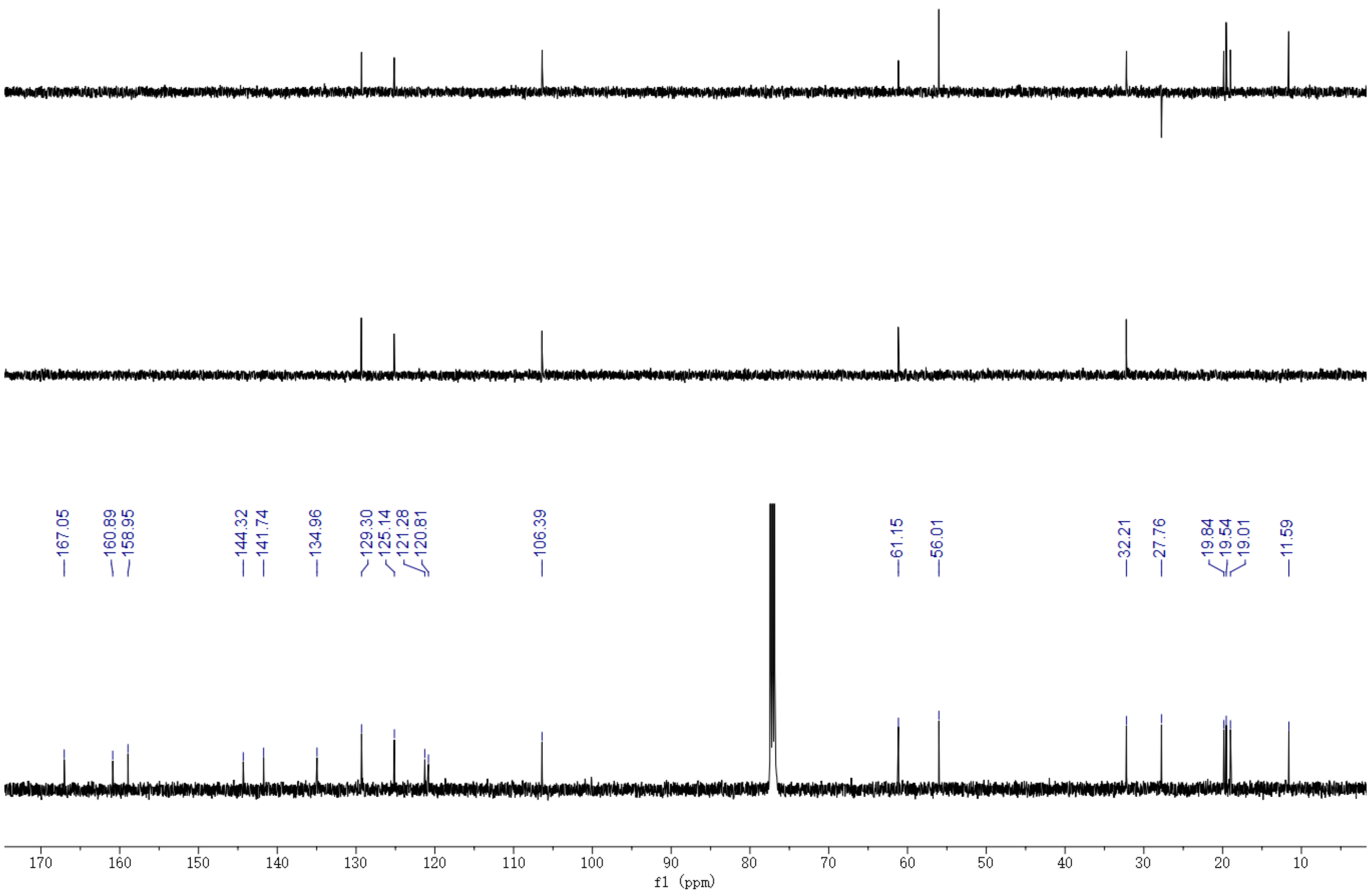

Figure S11. COSY spectrum of compound **2**.

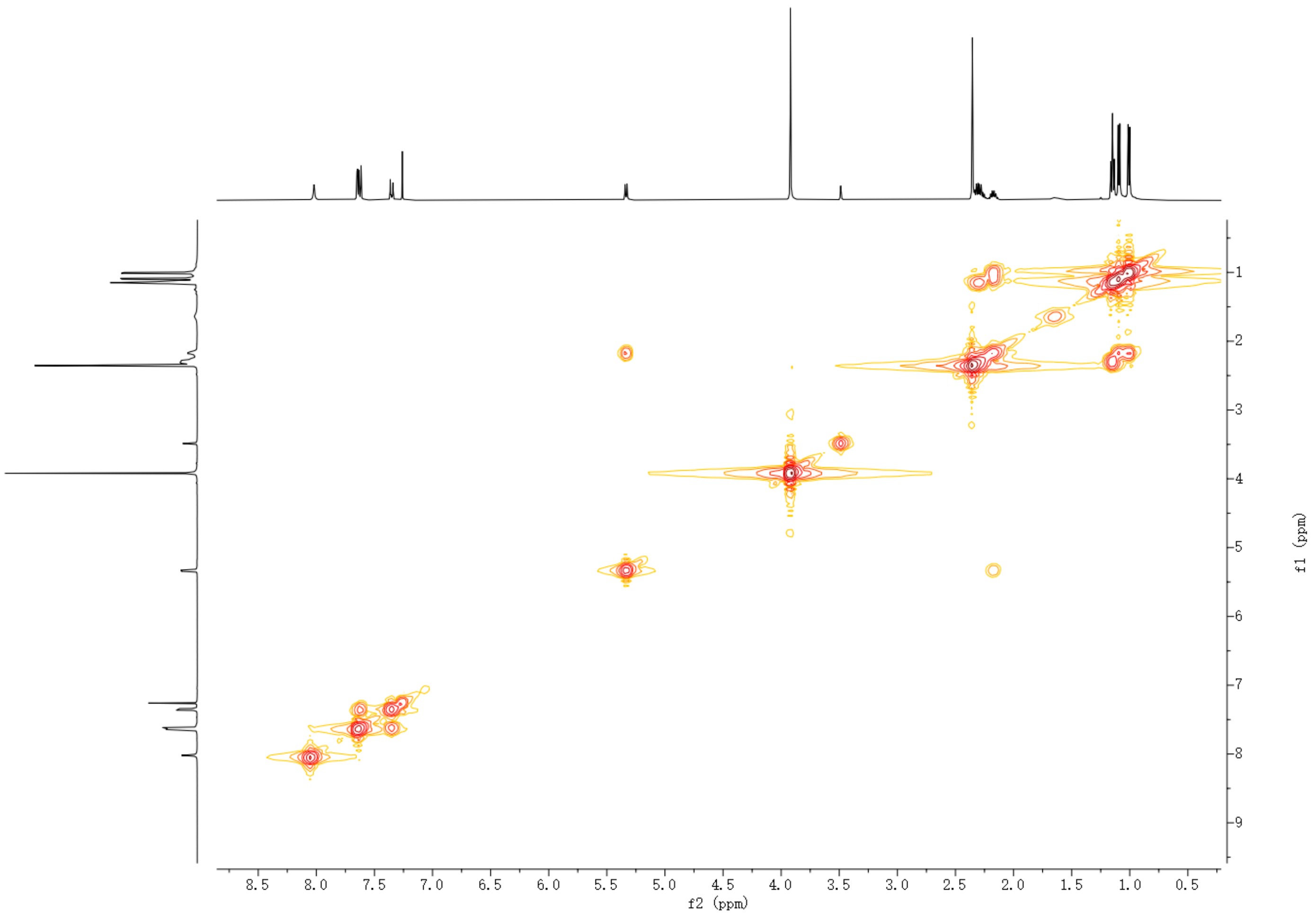

Figure S12. HSQC spectrum of compound **2**.

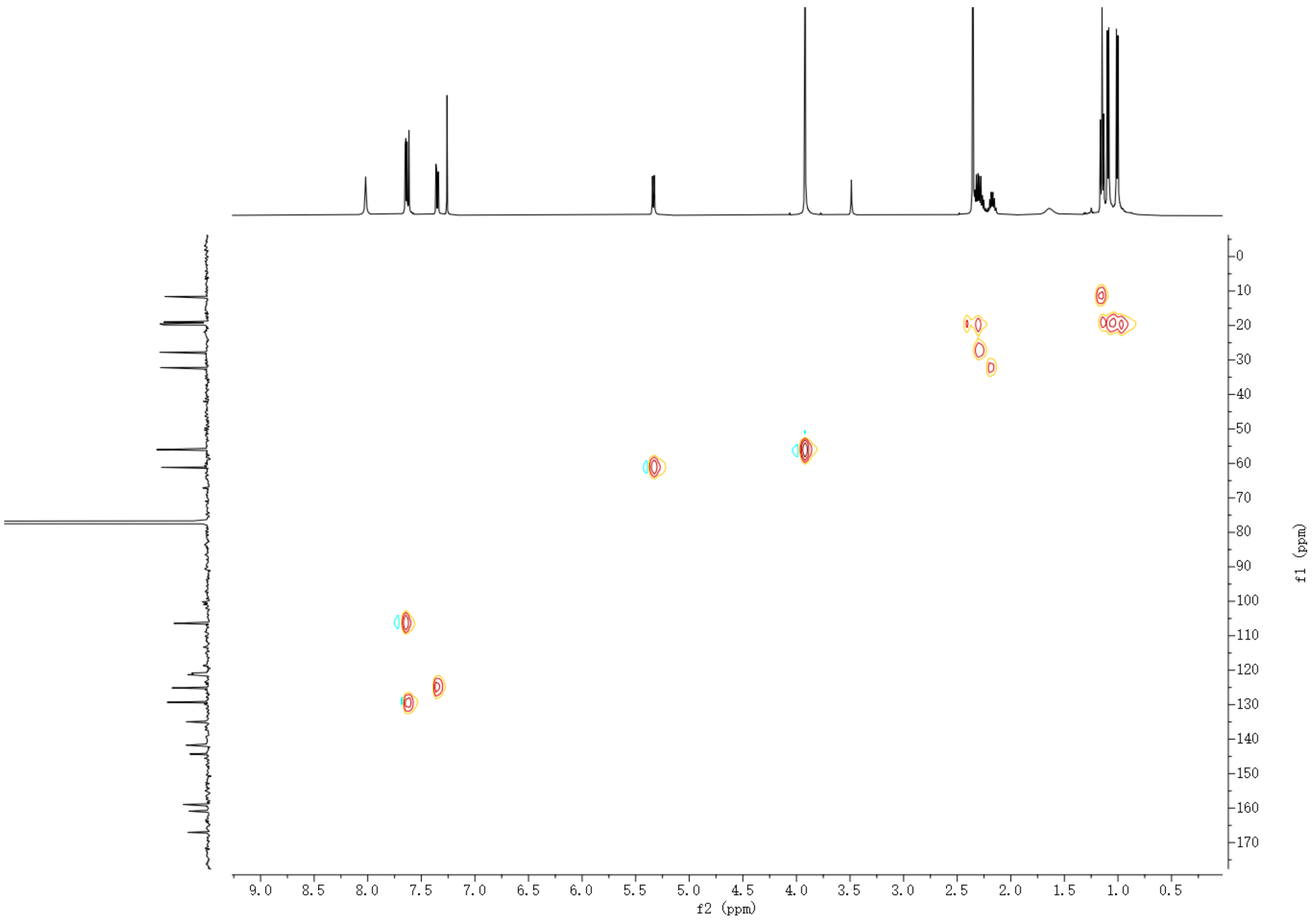

Figure S13. HMBC spectrum of compound **2**.

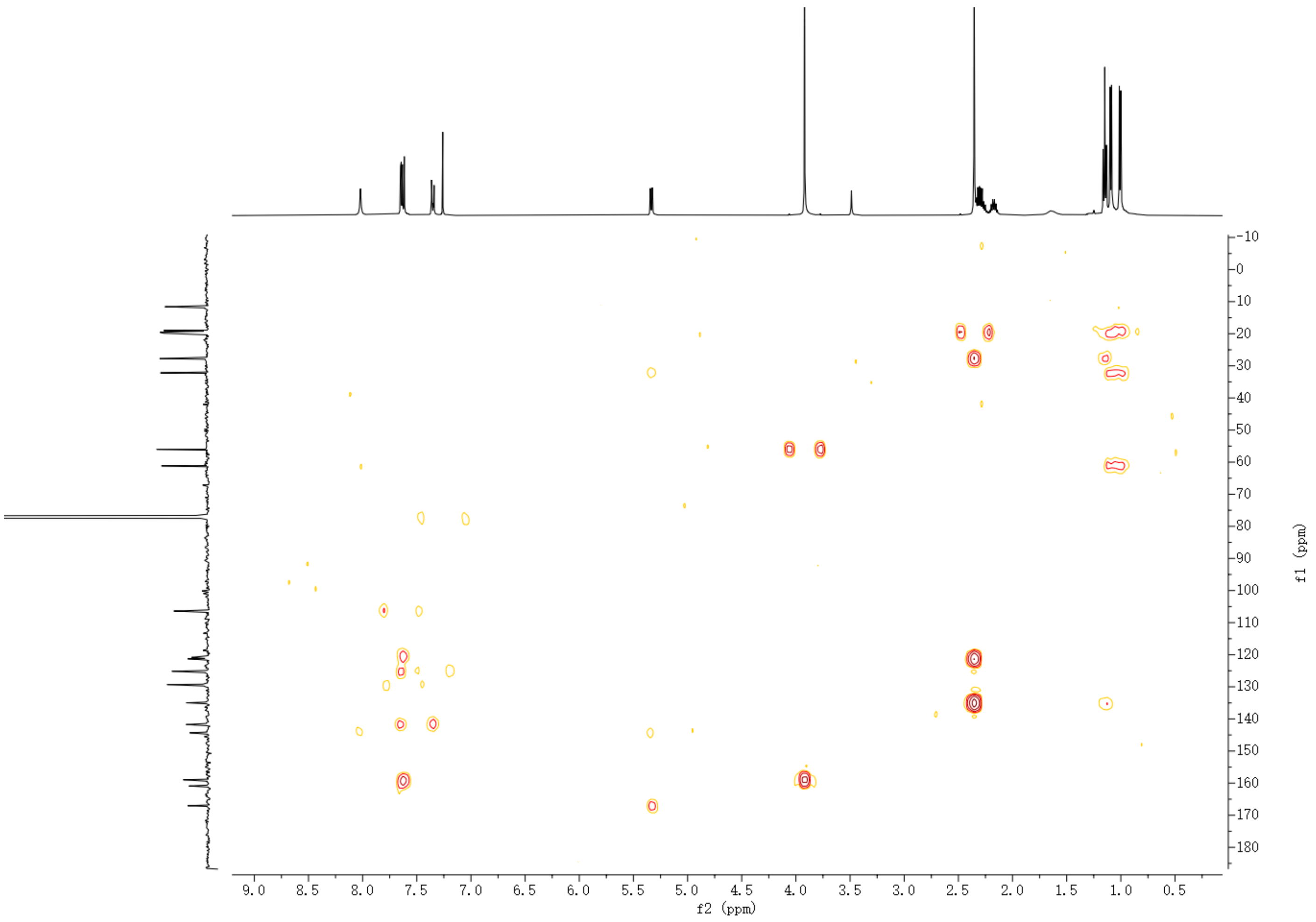

Figure S14. NOESY spectrum of compound **2**.

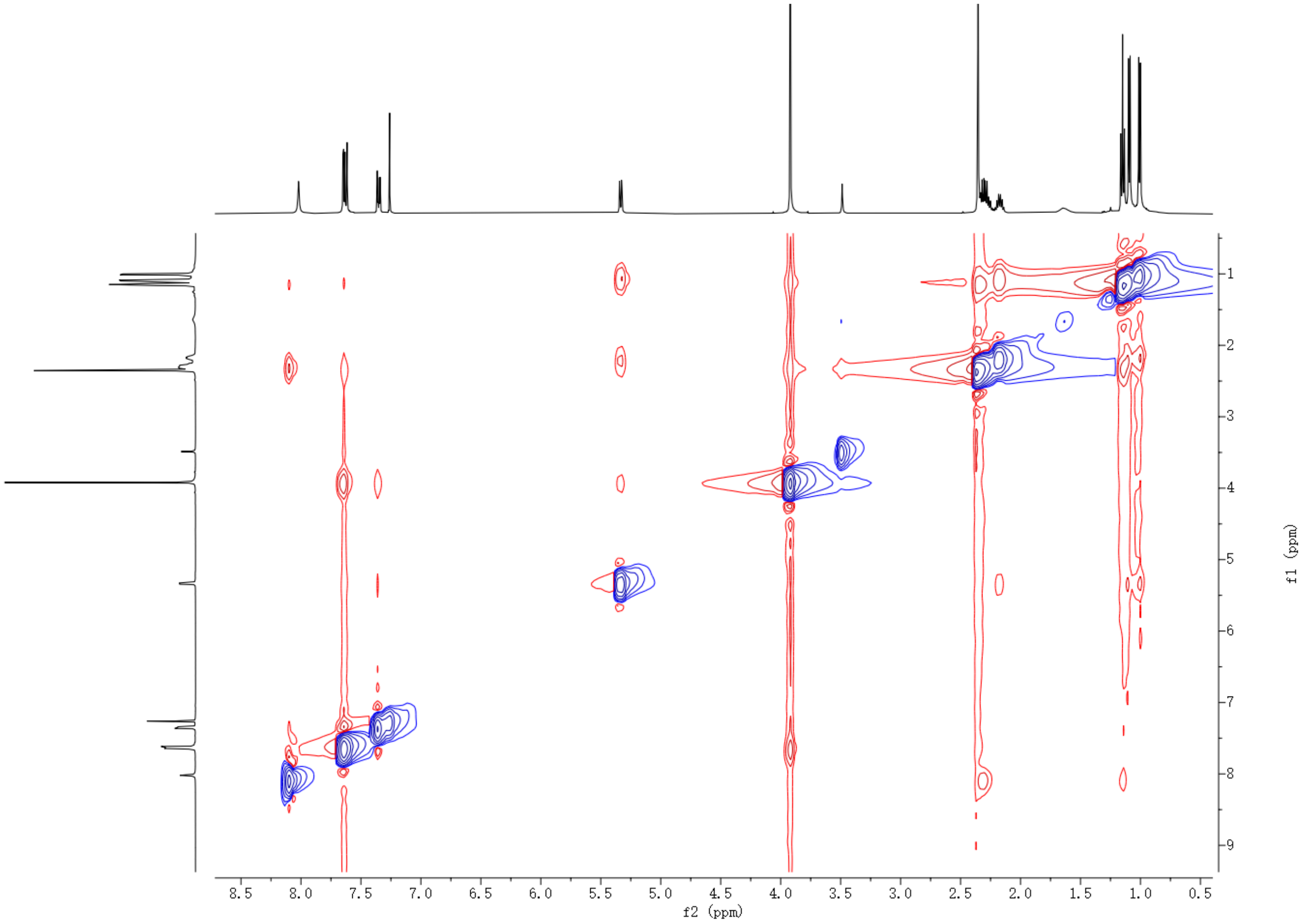

Figure S15. ^{1}H NMR (500 MHz, DMSO-d_6) spectrum of compound **3**.

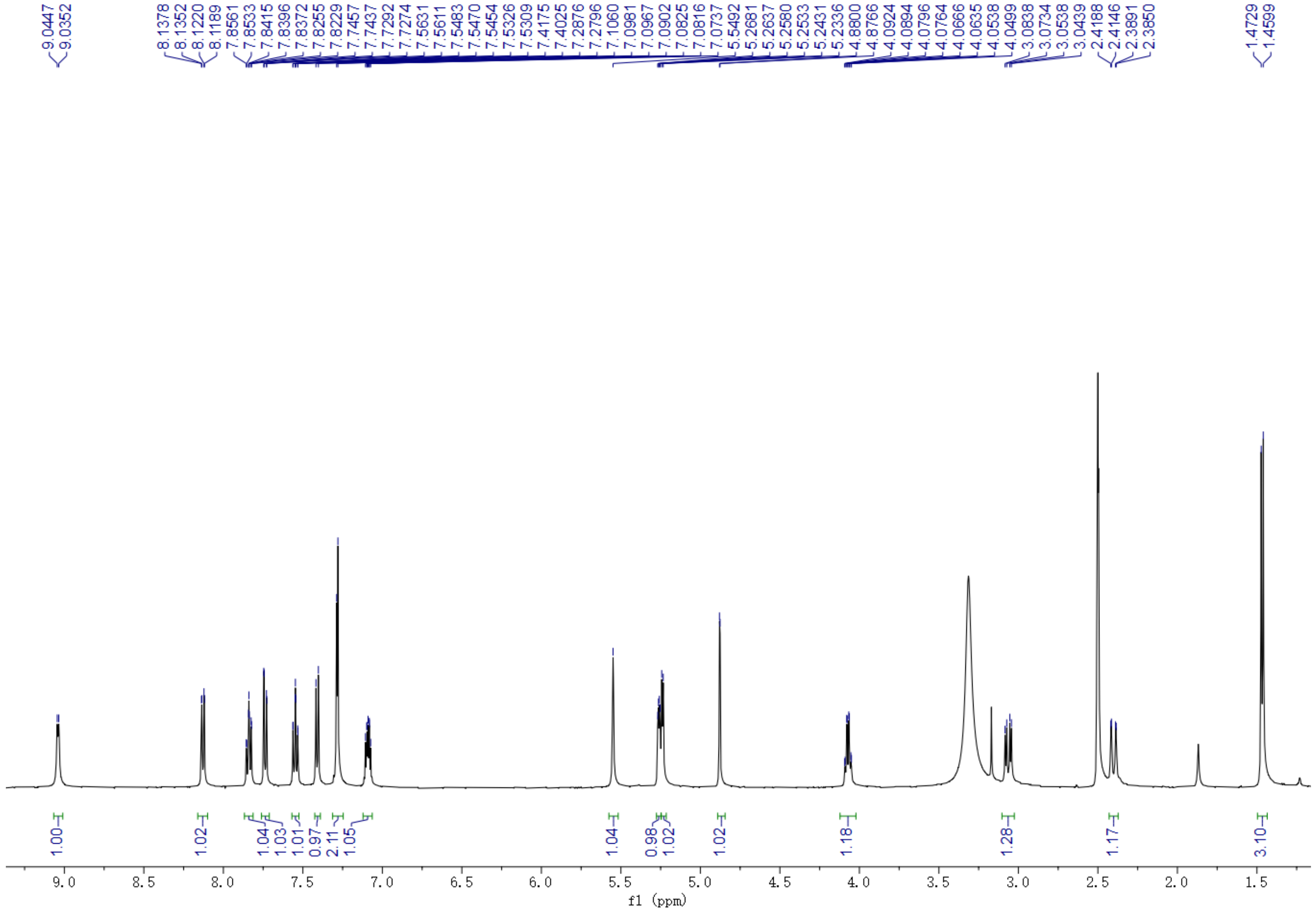

Figure S16. ^{13}C NMR (125 MHz, DMSO-d_6) and DEPT spectra of compound **3**.

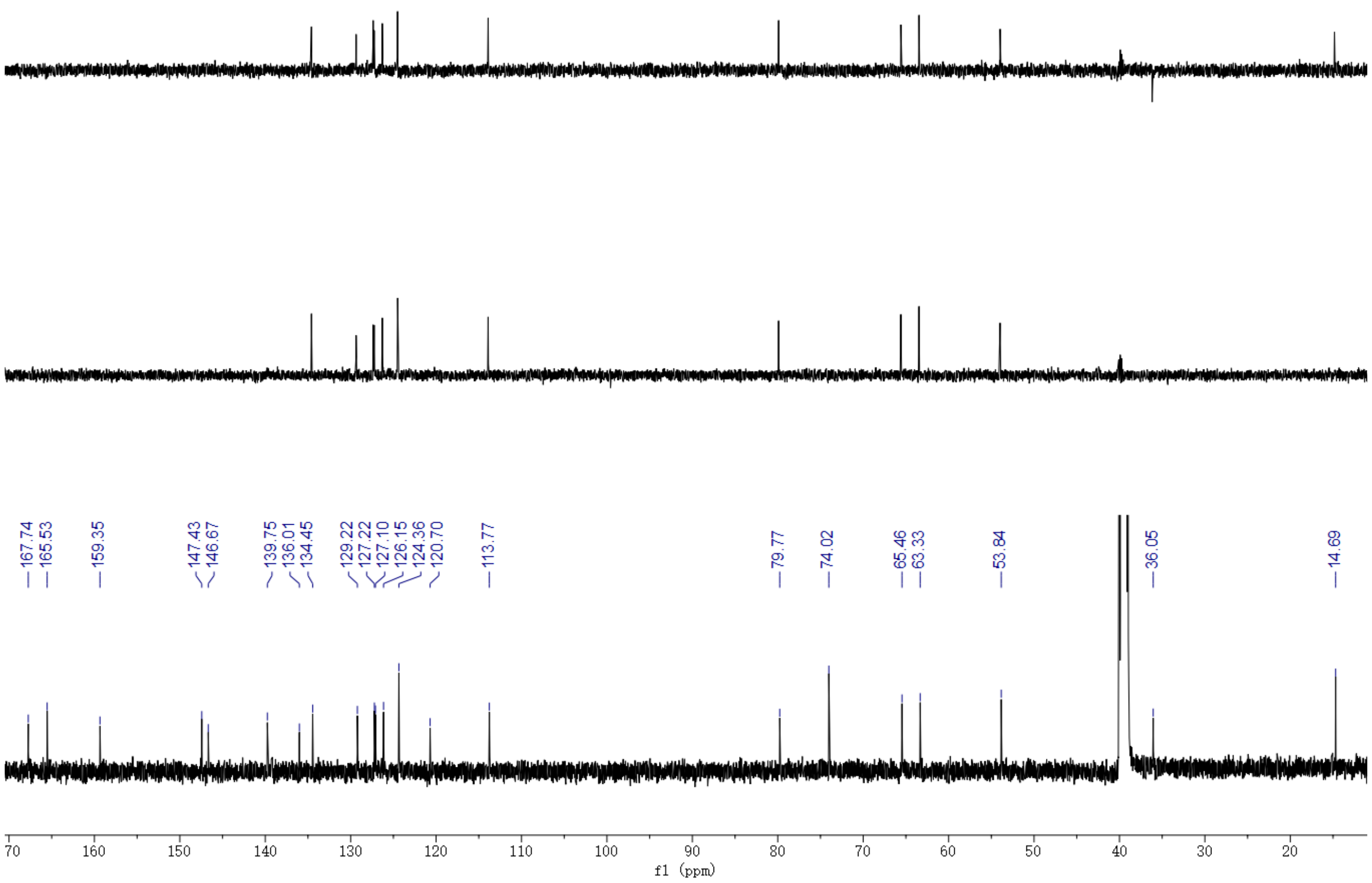

Figure S17. HRESI mass spectrum of compound **4**.

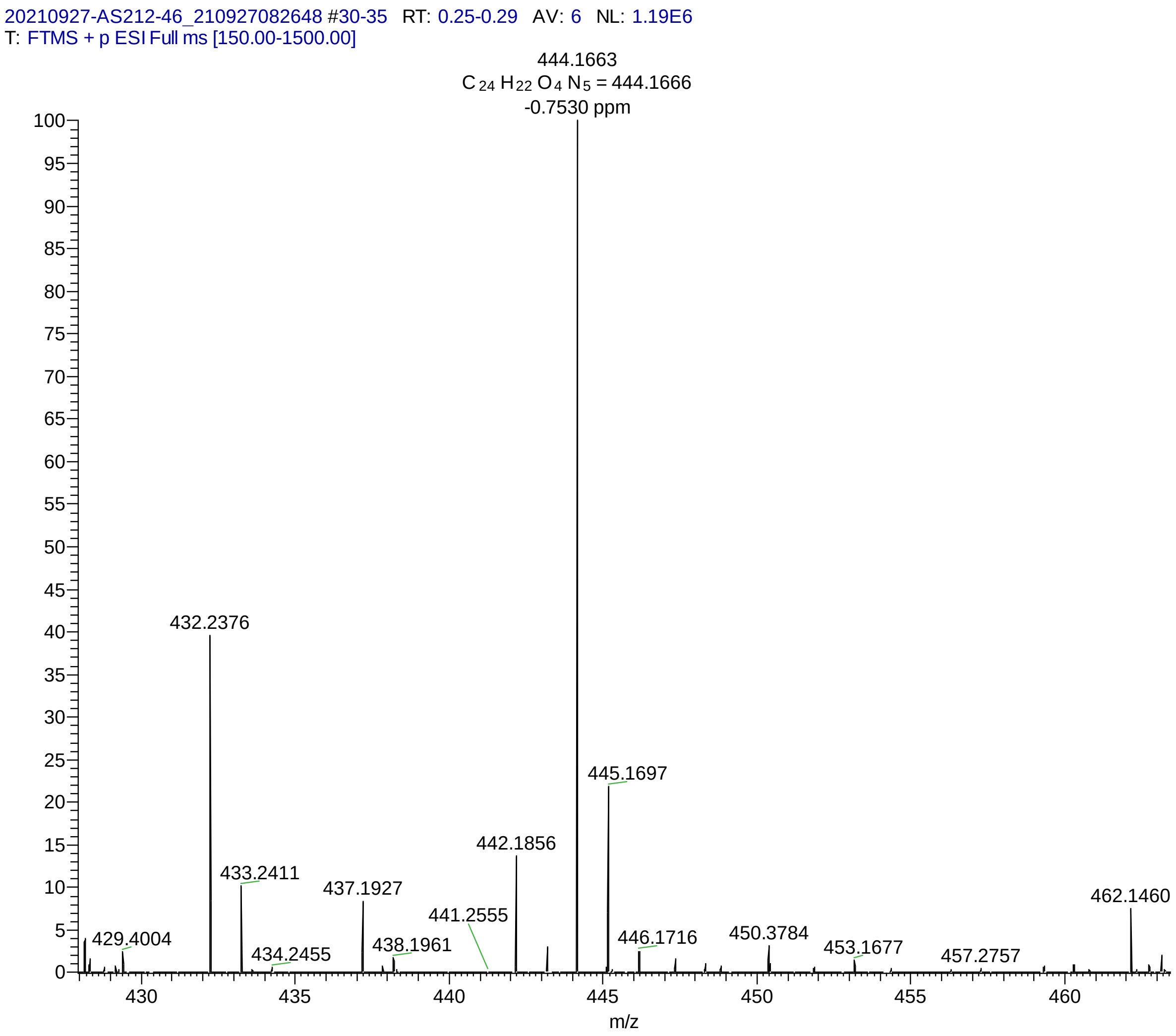

Figure S18. ^{1}H NMR (500 MHz, DMSO-d_6) spectrum of compound **4**.

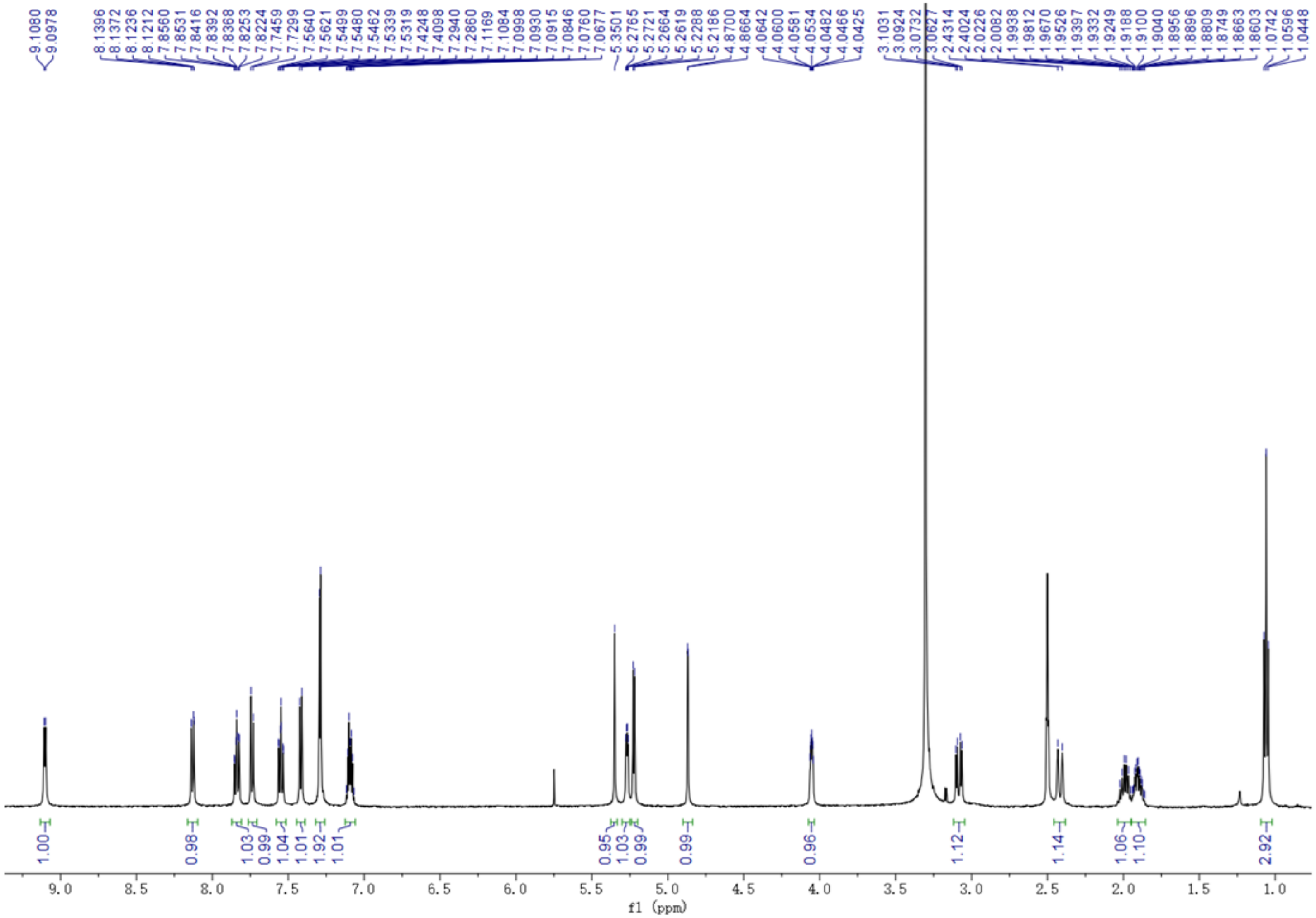

Figure S19. ^{13}C NMR (125 MHz, DMSO-d_6) and DEPT spectra of compound **4**.

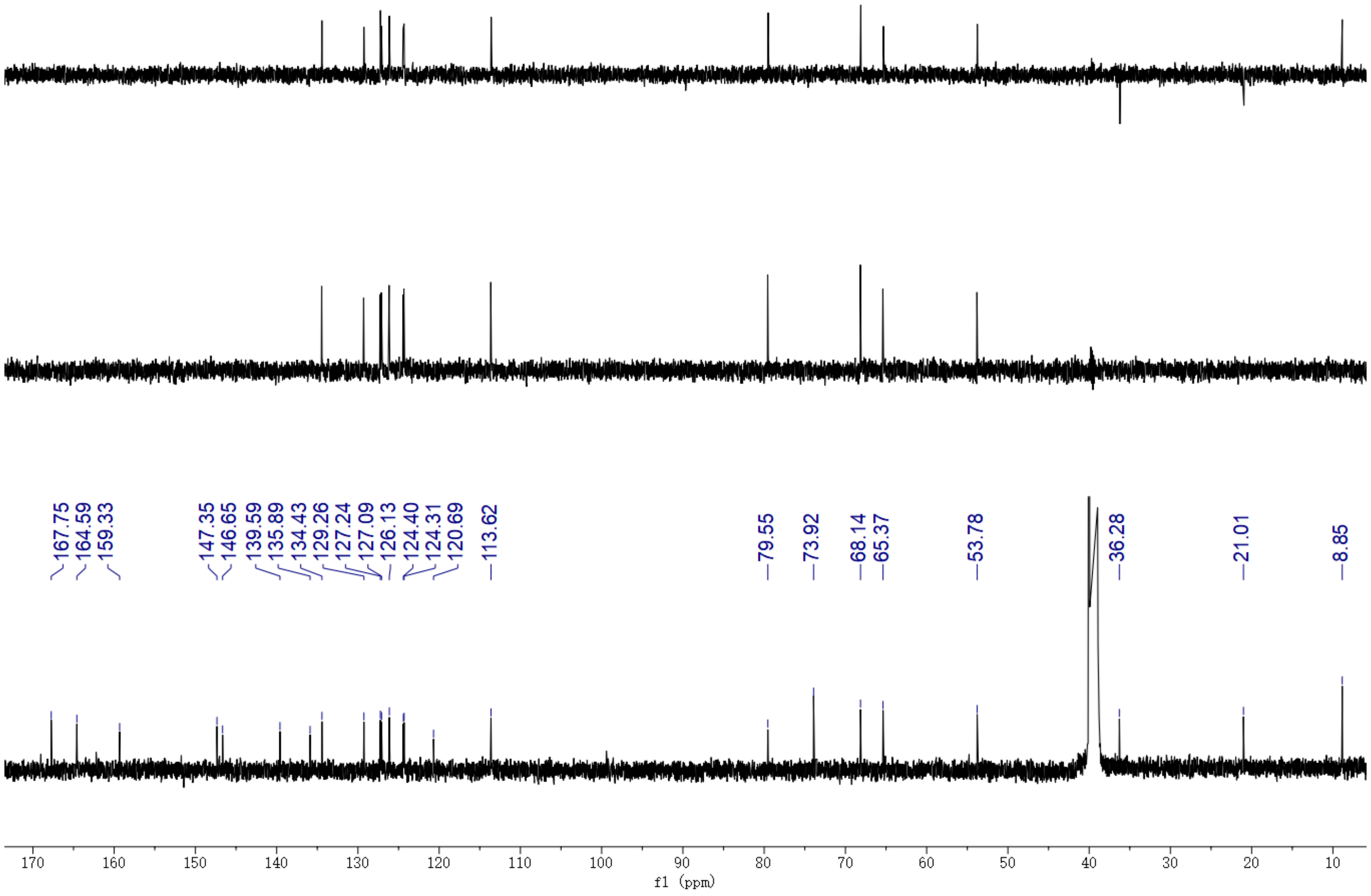

Figure S20. COSY spectrum of compound **4**.

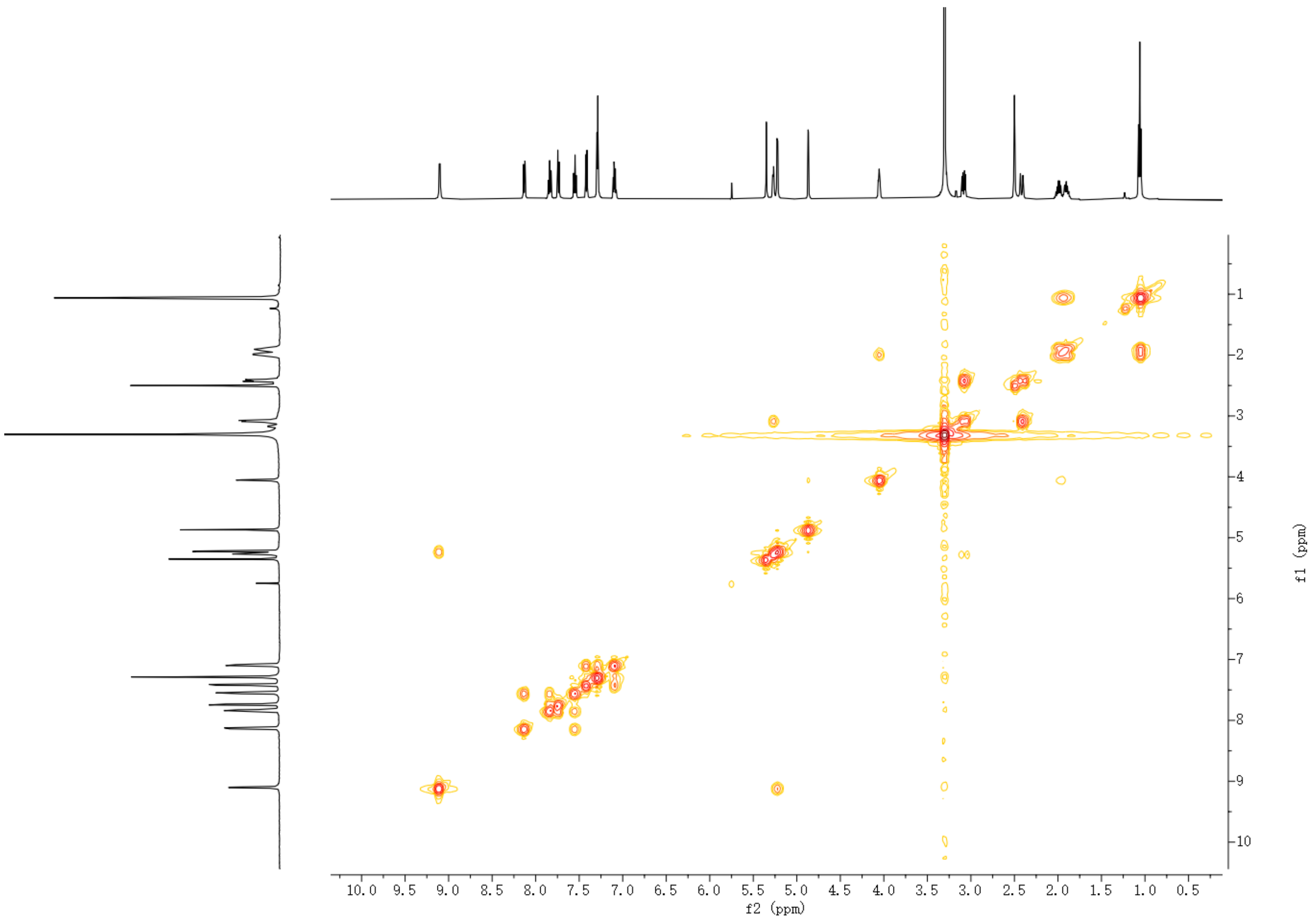

Figure S21. HSQC spectrum of compound **4**.

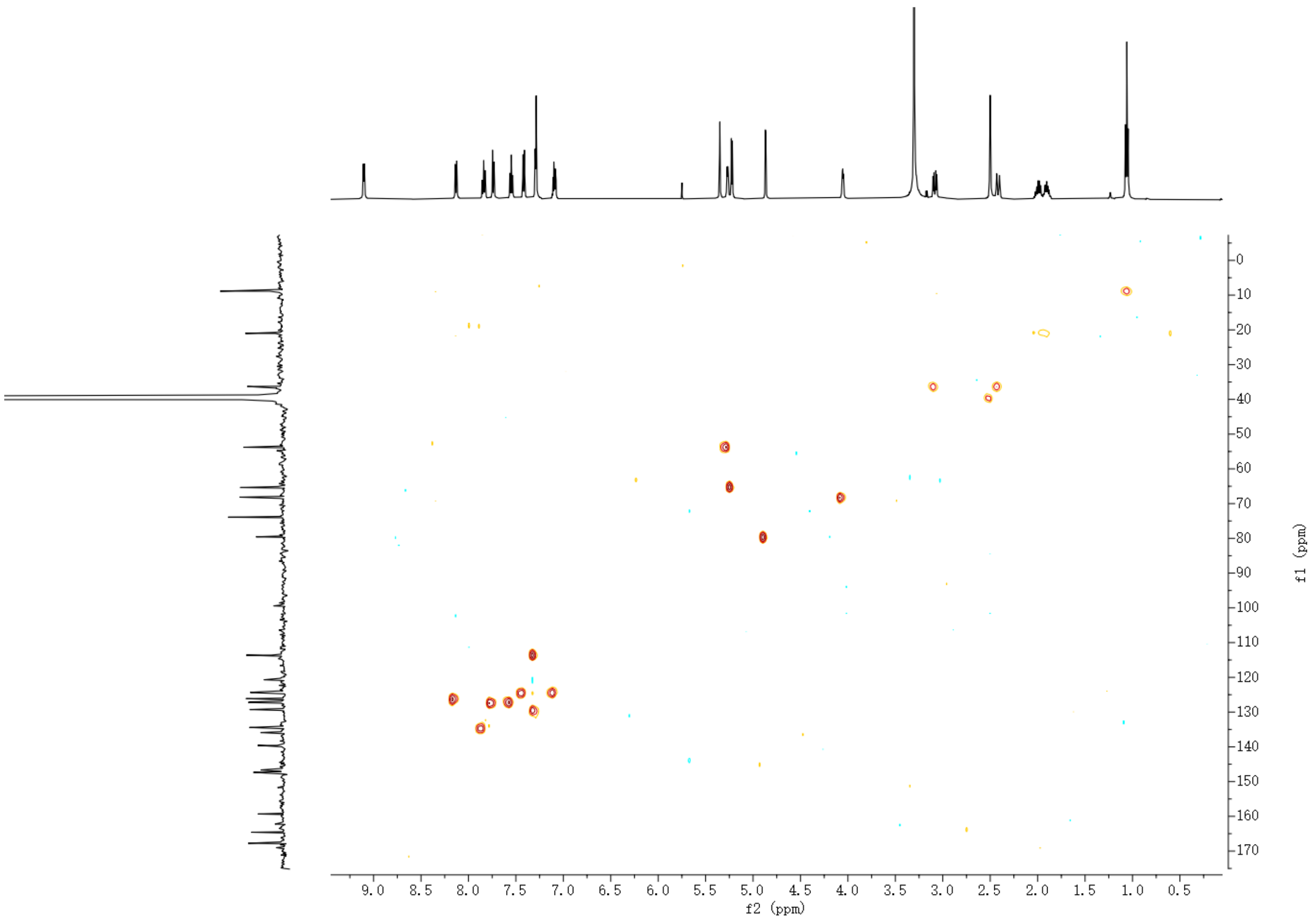

Figure S22. HMBC spectrum of compound **4**.

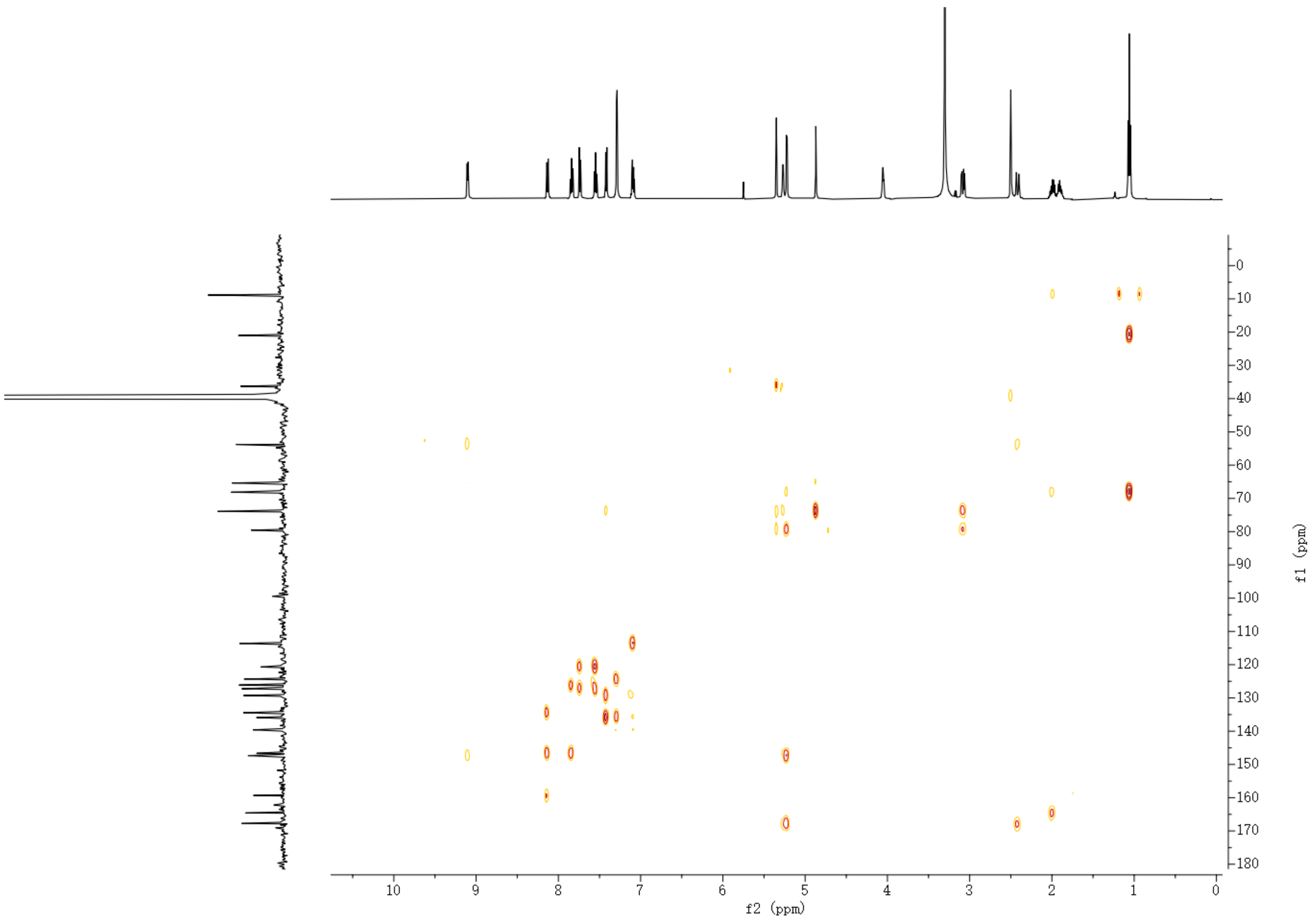

Figure S23. NOESY spectrum of compound **4**.

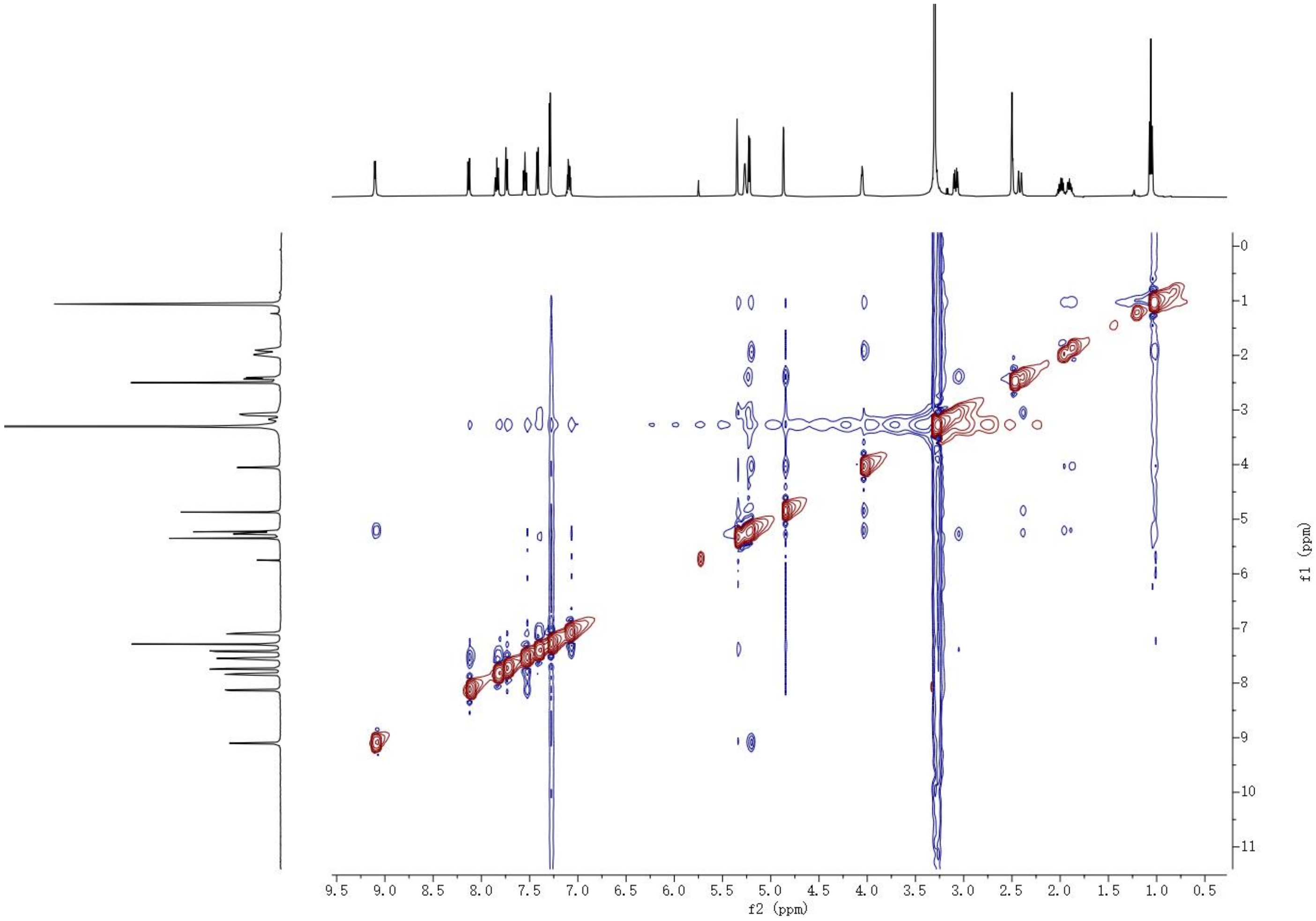

Figure S24. Crystal packing of compound **1** at 297(2) K.

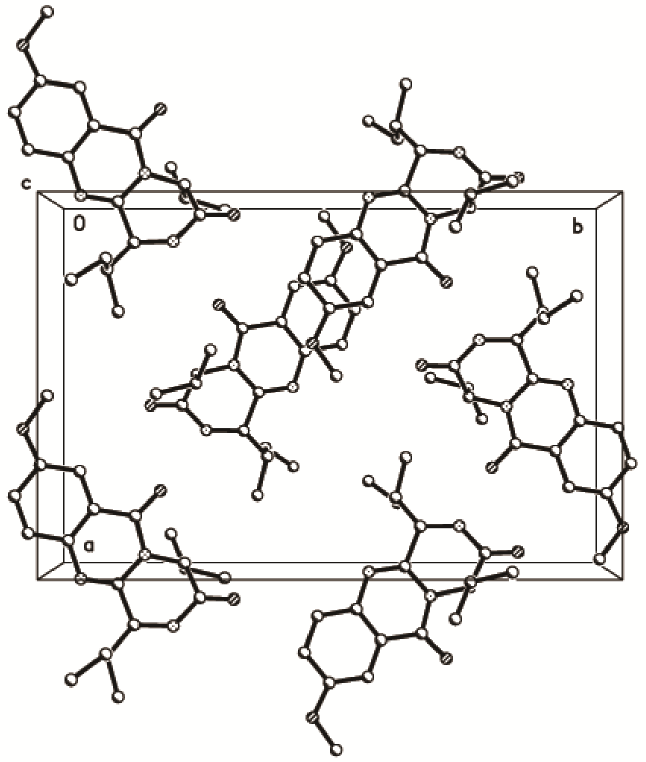

Figure S25. Crystal packing of compound **3** at 297(2) K.

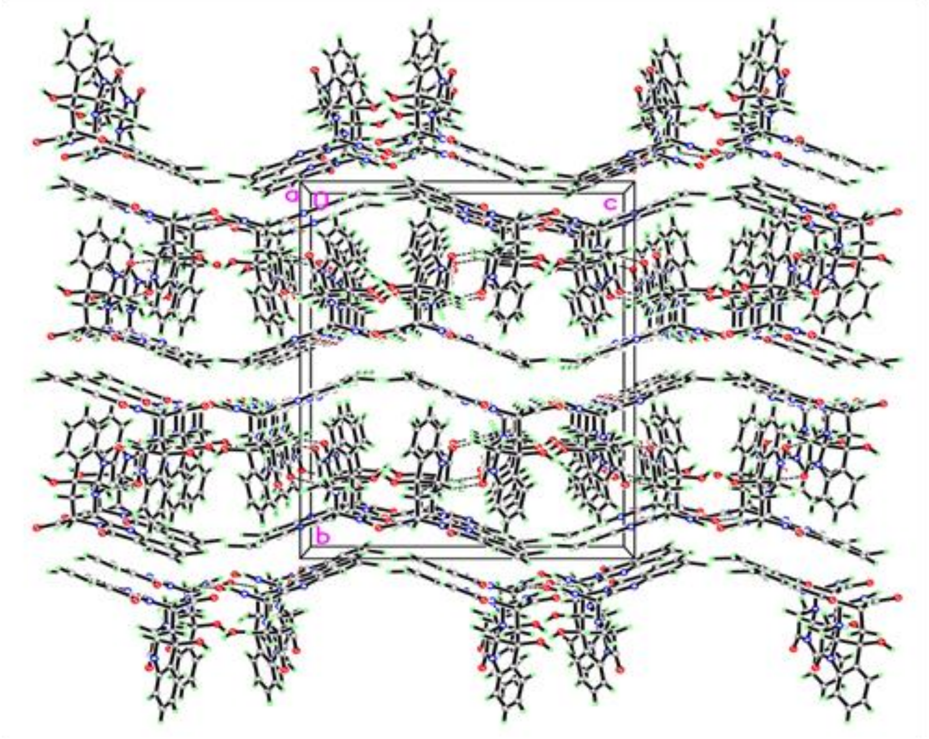

Figure S26. HPLC analysis of mycelia extract, broth extract, and compounds **1**−**12** of *Aspergillus versicolor* AS-212.

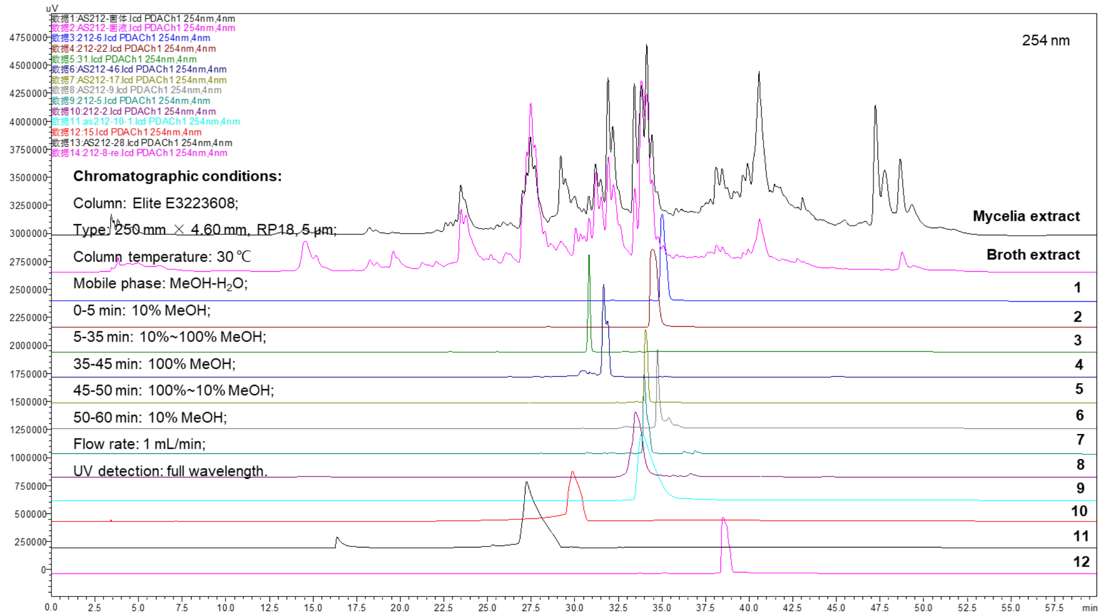

Figure S27. Experimental and calculated ECD spectra of compound **2** at the CAM-B3LYP/TZVP level.

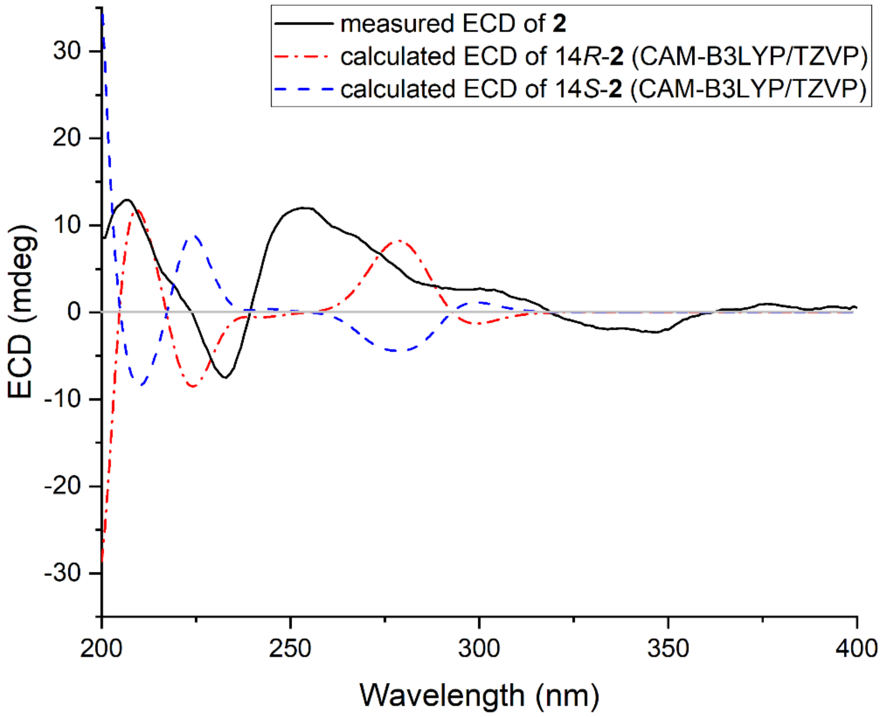

Table S1. Crystal data and structure refinement for compounds **1** and **3**.

Identification code	compound **1**	compound **3**
Empirical formula	$C_{19}H_{25}N_3O_3$	$C_{48}H_{36}N_{10}O_9S_2$
Formular weight	343.42	960.99
Temperature/K	293(2)	299(2)
Wavelength	1.54178	1.54178
Crytal system, space group	Orthorhombic, $P2_12_12_1$	Orthorhombic, $C222_1$
Unit cell dimensions	a = 13.2384(3) Å, alpha = 90 deg. b = 19.9347(4) Å, beta =90 deg. c = 6.8103(2) Å, gamma = 90 deg.	a = 9.4022(11) Å, alpha = 90 deg. b = 25.878(4) Å, beta =90 deg. c = 19.112(2) Å, gamma = 90 deg.
Volume/ $Å^3$	1797.26(8)	4650.2(11)
Z, Calculated density	4, 1.269 g/cm^3	4, 1.373 g/cm^3
Absorption coefficient/ mm^{-1}	0.702	1.612
F(000)	736	1992
Crystal size/mm^3	0.35 × 0.33 × 0.30	0.20 × 0.18 × 0.15
Theta range for data collection/°	13.38 to 132.08	11.512 to 136.928
Limiting indices	-15 ≤ h ≤ 14, -23 ≤ k ≤ 11,<br>-8 ≤ l ≤ 7	-11 ≤ h ≤ 11, -30 ≤ k ≤ 28, -22 ≤ l ≤ 23
Reflections collected/unique	4007/2826<br>[R_{int} = 0.0151, R_{sigma} = N/A]	21138/4267<br>[R_{int} = 0.0468, R_{sigma} = 0.0334]
Data/restraints/parameters	2826/0/231	4267/0/301
Goodness-of-fit on F^2	1.060	1.367
Final R indices [I>2sigma(I)]	R_1= 0.0379, wR_2 = 0.0989	R_1 = 0.0951, wR_2 = 0.2851
R indices (all data)	R_1 = 0.0411, wR_2 = 0.1014	R_1 = 0.1012, wR_2 = 0.2969
Absolute structure parameter	0.0(2)	0.145(12)
Extinction coefficient	n/a	n/a
Largest diff. Peak and hole	0.13 and -0.19 e A^-3	0.49 and -2.49 e A^-3

Table S2. Calculated specific rotation values at 589.44 nm for the enantiomers 14*R*-**2** and 14*S*-**2** at the CAM-B3LYP/TZVP level.

**Specific rotation calculation**	**CAM-B3LYP/TZVP**
14*R*-**2**	+59.8
14*S*-**2**	–59.8

Table S3. ^{1}H and ^{13}C NMR spectroscopic data for compound **3**.

no.	**3**[a]	
	δ_C[a]	δ_H[b] (*J* in Hz)
1	167.7, C	
2		9.04, d, (4.8)
3	65.5, CH	5.24, d, (4.8)
4	147.4, C	
6	146.7, C	
7	127.1, CH	7.74, dd, (8.3, 1.0)
8	134.5, CH	7.84, ddd, (8.3, 7.3, 1.4)
9	127.2, CH	7.55, ddd, (8.0, 7.3, 1.0)
10	126,2, CH	8.13, dd, (8.0, 1.4)
11	120.7, C	
12	159.4, C	
14	53.8, CH	5.26, dd, (5.1, 2.3)
15	36.1, CH_2	3.06, dd, (14.9, 5.3)
		2.40, dd, (14.9, 2.3)
17	74.0, C	
18	79.8, CH	4.88, d (1.7)
20	63.3, CH	4.07, dq (6.6, 1.7)
21	165.5, C	
23	136.0, C	
24	113.8, CH	7.28, overlap
25	129.2, CH	7.28, overlap
26	124.4, CH	7.09, m
27	124.4, CH	7.41, d, (7.5)
28	139.8, C	
29	14.7, CH_3	1.47, d (6.5)
17-OH		5.55, br s

[a]Measured at 125 MHz in DMSO-d_6. [b]Measured at 500 MHz in DMSO-d_6.
